# Supplementary material for: Patterned invagination prevents mechanical instability during gastrulation
Source: Nature. 2025 Sep 3;646(8085):627–36. doi: 10.1038/s41586-025-09480-3 (PMC12527948; doi:10.1038/s41586-025-09480-3)
Supplement: Supplementary file 1 — Supplementary Figures 1–5, Supplementary Notes 1–7 and Supplementary Table 1 [file 41586_2025_9480_MOESM1_ESM.docx]

# Supplementary information

Patterned invagination prevents mechanical instability during gastrulation

Bruno C. Vellutini^1,✉^, Marina B. Cuenca^1^, Abhijeet Krishna^1,2,3^, Alicja Szałapak^1,2,3^, Carl D. Modes^1,2,3^, and Pavel Tomancak^1,2,3,✉^

^1^ Max Planck Institute of Molecular Cell Biology and Genetics, Dresden, Germany
^2^ Center for Systems Biology Dresden, Dresden, Germany
^3^ Cluster of Excellence Physics of Life, Technische Universität Dresden, Dresden, Germany

^✉^ Correspondence: [Bruno C. Vellutini <vellutini@mpi-cbg.de>](mailto:vellutini@mpi-cbg.de), [Pavel Tomancak <tomancak@mpi-cbg.de>](mailto:tomancak@mpi-cbg.de)

## Supplementary Figure 1


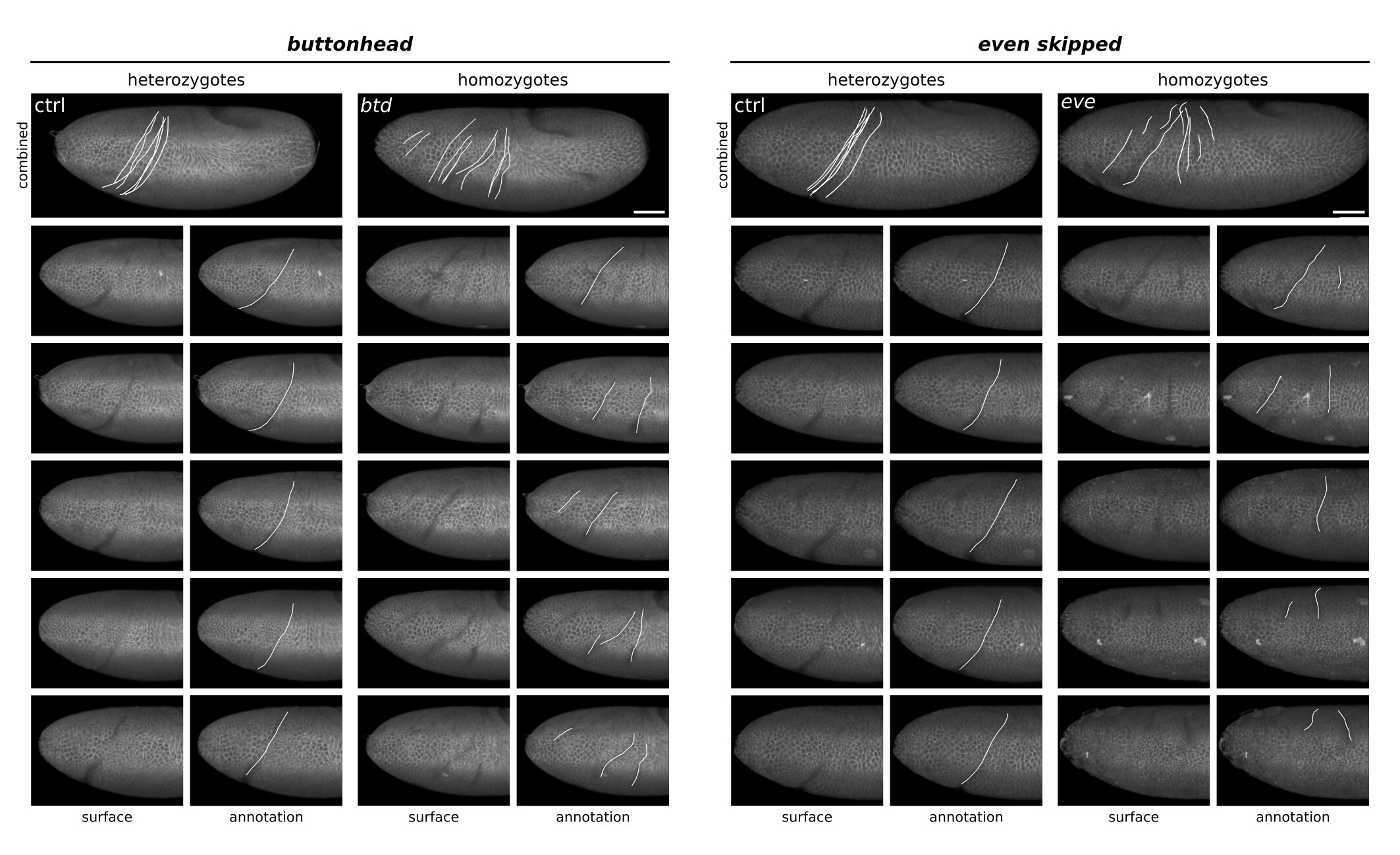


Supplementary Fig. 1: Individual variability of ectopic folding in *btd* and *eve* mutants. The cephalic furrow and ectopic folds are traced in white lines. The combined views illustrate the position of folding overlaid over a single embryo after embryo registration. Scale bars = 50 µm.

## Supplementary Figure 2


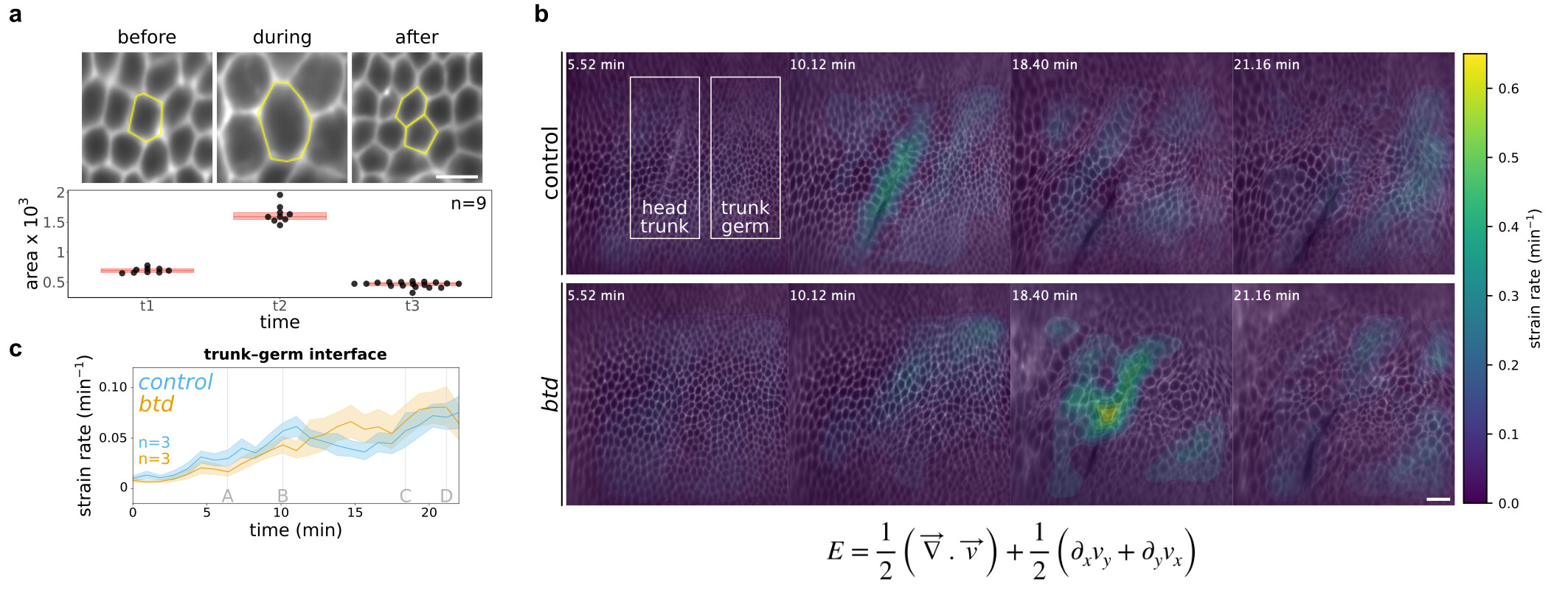


Supplementary Fig. 2: Complementary analyses of mitotic expansions and strain rate. **a**, Increase in the apical area of individual cells within mitotic domains. A dividing cell increases its apical area 2.4 times during mitotic rounding. The individual daughter cells retain 66% of the parent apical area. When summed, the apical area of the two daughter cells occupies 1.3 times the original apical area of their parent cell. Scale bar = 10 µm. Data from 9 tracked cells on a single *btd* heterozygote embryo. **b**, Strain rate analysis in *btd* mutants. Cropped region of cartographic projections of *btd* sibling controls (top, n=3) and homozygote embryos (bottom, n=3). The membrane marker (Gap43-mCherry) is overlayed with a heatmap indicating the regions of increased strain rate in the tissue. The value is the sum of isotropic and anisotropic strain rates obtained through a particle image velocimetry analysis. We used the strain rates in the regions outlined as head–trunk and trunk–germ to generate the plot in Fig. [2](#fig%3Amitogerm-experiments)g. Scale bar ≈ 20 µm. **c**, Strain rate at the trunk–germ region in *btd* heterozygotes (n=3) and homozygotes (n=3). Lines A–D represent exact frames from **b**. Measurements combine isotropic and anisotropic strain rates.

## Supplementary Figure 3


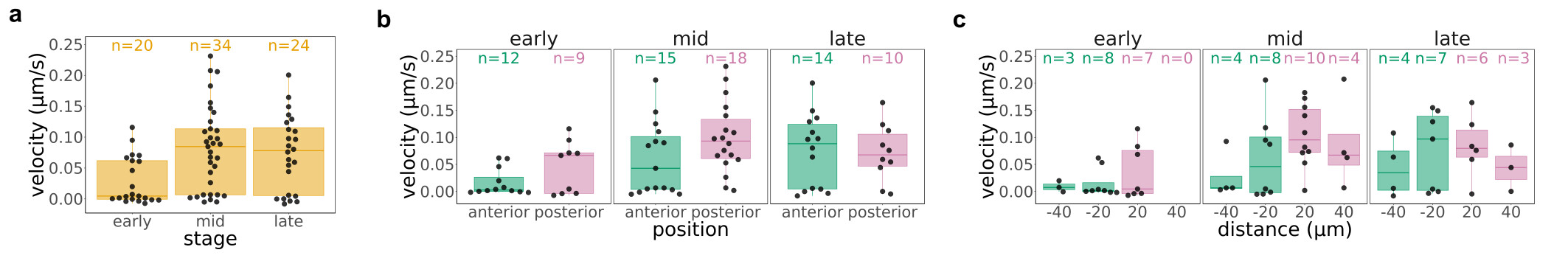


Supplementary Fig. 3: Laser ablations around initiator cells in wildtype embryos. The plots show the recoil velocity of cell membranes after laser ablation. **a**, Recoil velocity aggregated by early (n=20), mid (n=34), and late (n=24) stage 6. **b**, Recoil velocity split into cuts anterior and posterior to the initiator cells in early (n=12, n=9), mid (n=15, n=18), and late (n=14, n=10) stage 6. **c**, Average recoil velocity by 20 µm distance classes for early anterior (n=3, n=8) and posterior (n=7, n=0), mid anterior (n=4, n=8) and posterior (n=10, n=4), and late anterior (n=4, n=7) and posterior (n=6, n=3) stage 6.

## Supplementary Figure 4


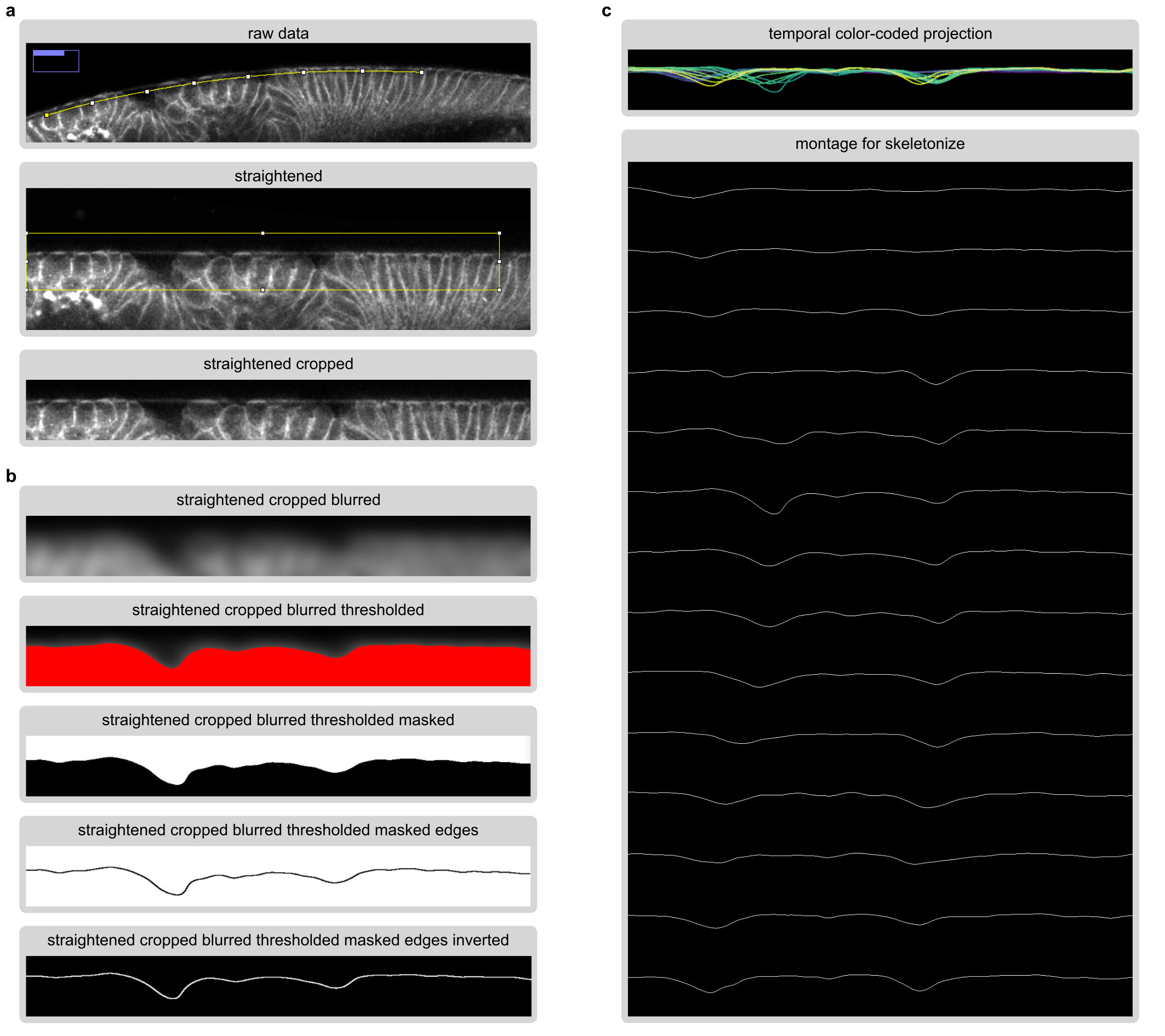


Supplementary Fig. 4: Image processing pipeline for the tortuosity analysis in cauterized mutants. **a**, We acquired a single slice in profile view of the head–trunk epithelium. First, we straightened the epithelial monolayer along the curvature of the vitelline envelope using the Straighten tool in ImageJ. We then cropped a window to standardize the size of the analyzed area for all embryos. **b**, Then, we applied a Gaussian blur, which allows capturing the deformations in the epithelium caused by the ectopic folds after thresholding. We create a mask and detect edges and invert to retain a single pixel line corresponding to the outline of the epithelium. The image is inverted for downstream processing. **c**, We applied temporal color-coded projections to visualize the epithelial dynamics over time and created a montage with all timepoints to extract the length of the outline using the skeletonize plugin in ImageJ.

## Supplementary Figure 5


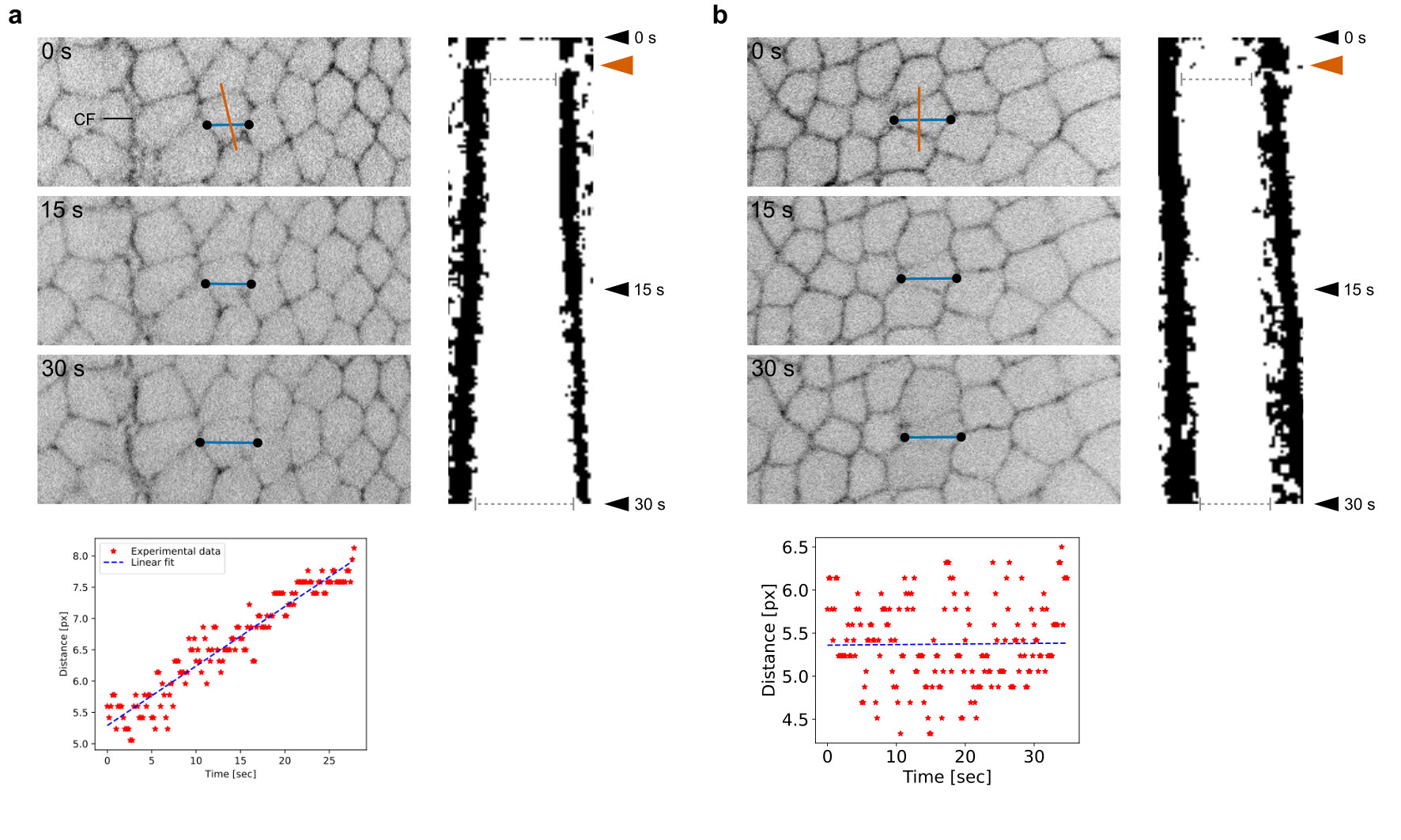


Supplementary Fig. 5: Image processing pipeline for the ablation analysis. **a**, Example of laser ablation near the cephalic furrow with the membrane signal (top left), the generated kymograph (right), and the linear fit over the distance between edges extracted from the kymograph (bottom left). The position of the laser cut is annotated in a vermilion line, the cell edges are marked in black circles, and the edge distances in a blue line. The distance between edges increases over time. **b**, Example of a laser ablation far from the cephalic furrow where the distance between edges does not increase over time.

## Supplementary Video 1


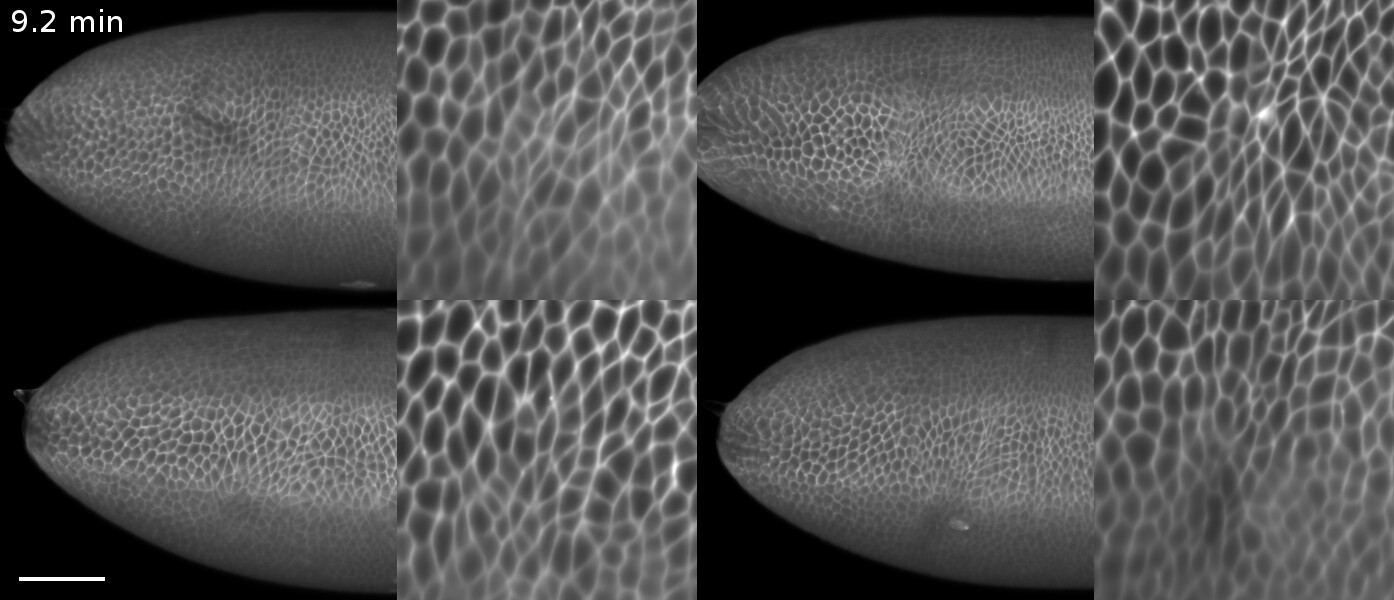


Supplementary Video 1: Reduced apical constriction in *btd* mutants. Lateral view (left) and cartographic projection (right) of the head–trunk interface in four individual *btd* mutants. Putative initiator cells (center) exhibit a reduced degree of apical constriction. The video is looped to highlight the changes in apical cell area. Frame rate = 10 fps. Scale bar = 50 µm.

## Supplementary Video 2


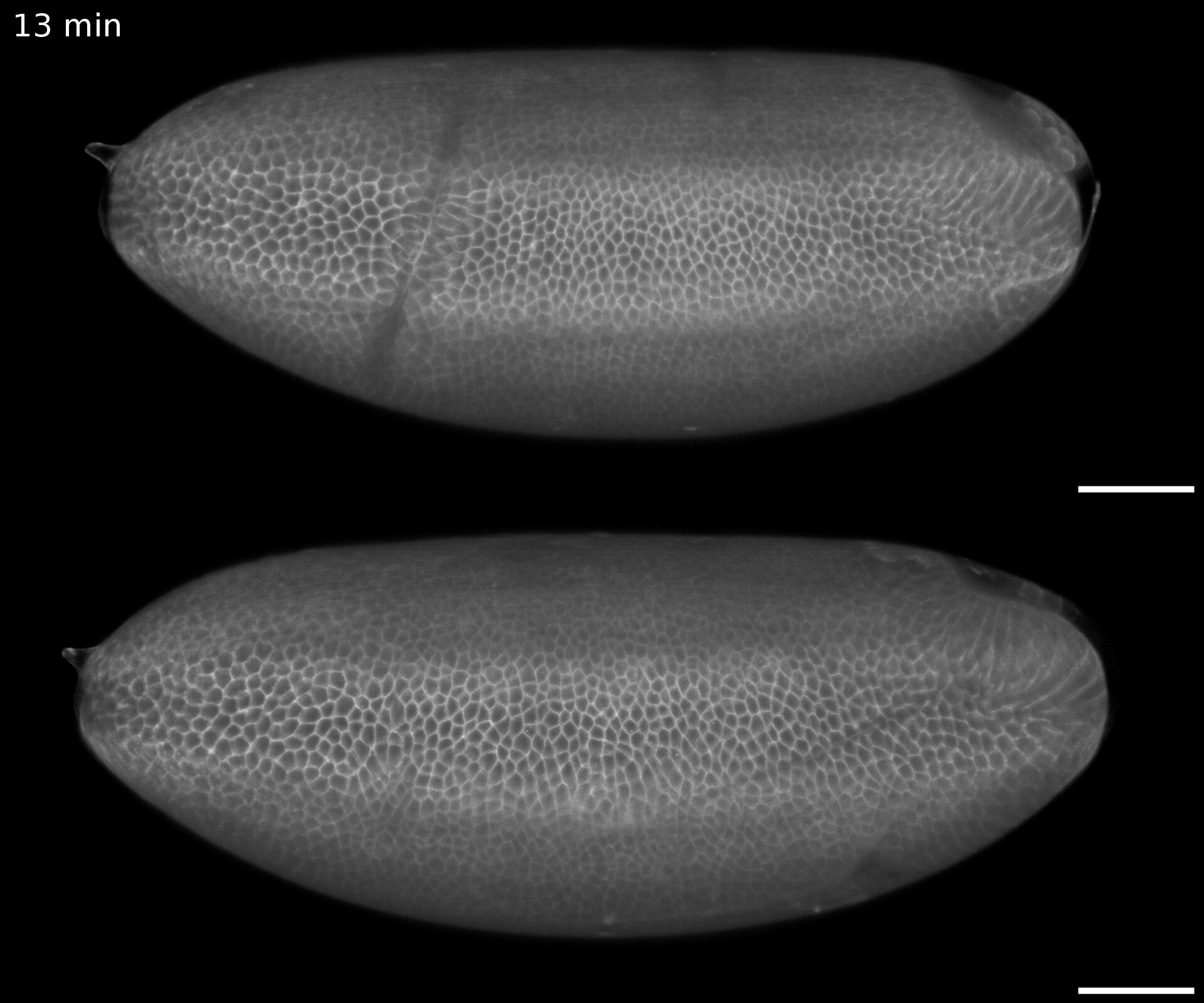


Supplementary Video 2: Lateral view of ectopic fold formation in *btd* mutant. The cephalic furrow forms normally in sibling controls (top) but is absent in *btd* mutants (bottom). In the mutant, no fold is present at the head–trunk interface until about 20 min, when a large ectopic fold appears and quickly unfolds at about 45 min. In the sibling control, the cephalic furrow remains partially invaginated for the period shown in the recording (about 110 min). Frame rate = 15 fps. Scale bars = 50 µm.

## Supplementary Video 3


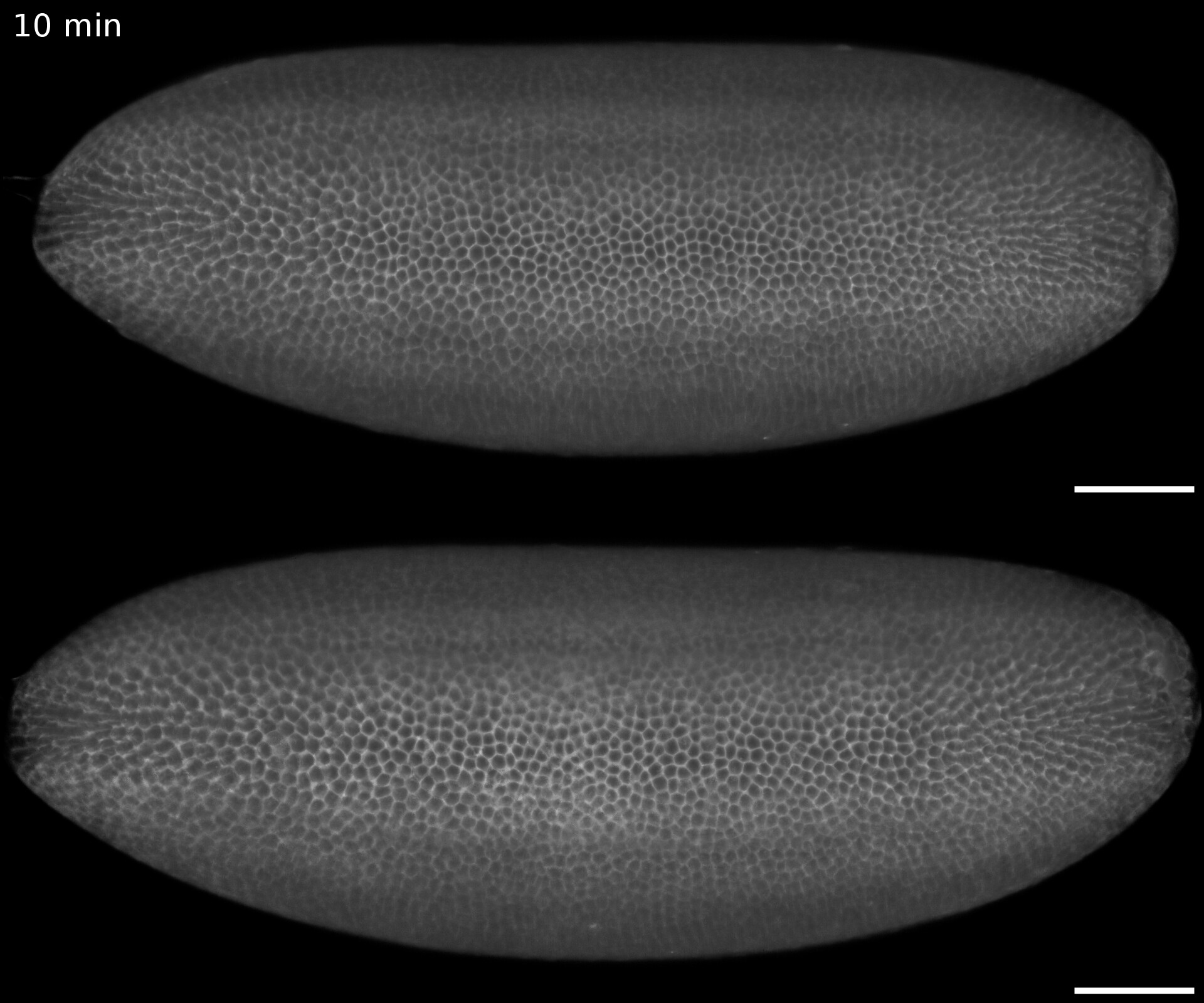


Supplementary Video 3: Lateral view of ectopic fold formation in *eve* mutant. The cephalic furrow forms normally in sibling controls (top) but is absent in *eve* mutants (bottom). There is no invagination at the head–trunk boundary at the onset of gastrulation, but an ectopic fold starts forming near the dorsal region as soon as the mitotic domains begin expanding around 24 min. The ectopic folds unfold almost entirely by the end of the recording (about 85 min). Additional ectopic folds appear in the trunk region. Frame rate = 10 fps. Scale bars = 50 µm.

## Supplementary Video 4


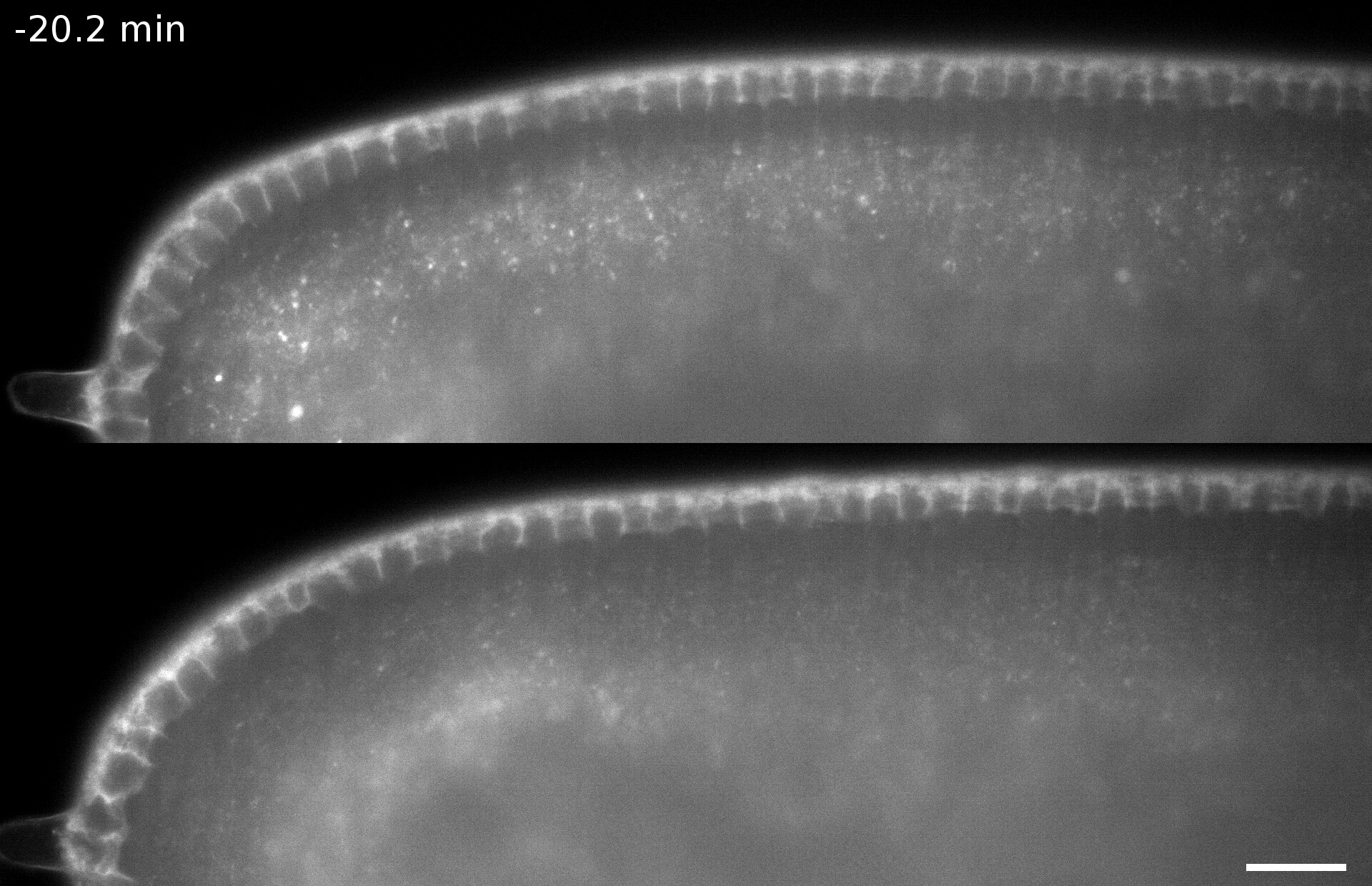


Supplementary Video 4: Profile view of ectopic fold formation in *btd* mutant. In sibling controls (top), the cephalic furrow initiates at the onset of gastrulation (1.5 min) and is fully invaginated when the cell divisions start (about 11 min). In *btd* mutants (bottom), no invagination initiates, but some embryos exhibit a bulging of the epithelium due to a residual apical constriction behavior (about 7 min). An ectopic fold forms at this position. Its morphology differs greatly from the cephalic furrow (see 10 min). Both the cephalic furrow and ectopic folds regress with the extension of the germ band. Frame rate = 10 fps. Scale bar = 20 µm.

## Supplementary Video 5


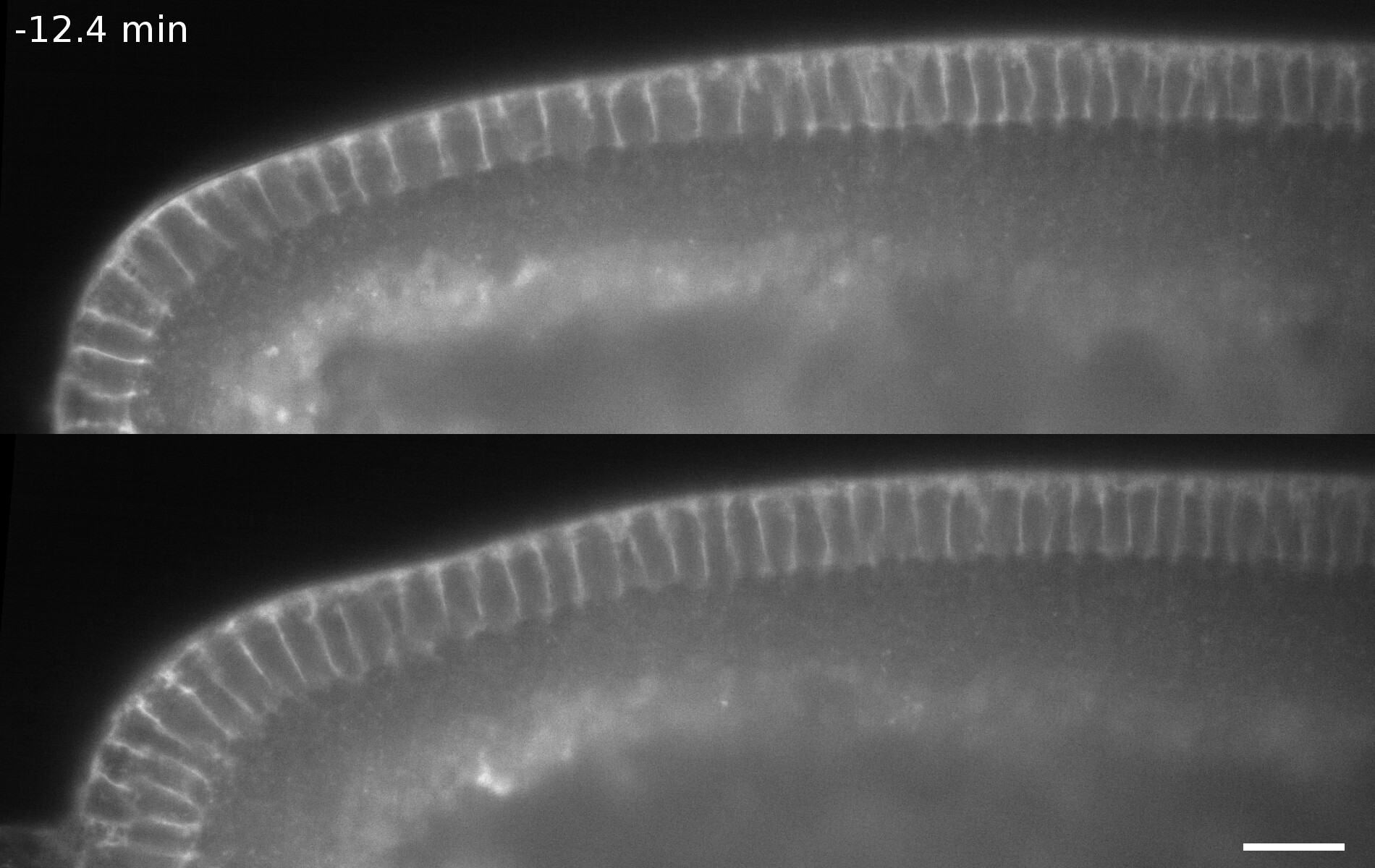


Supplementary Video 5: Profile view of ectopic fold formation in *eve* mutant. In sibling controls (top), the cephalic furrow initiates at the onset of gastrulation (1.8 min). In *eve* mutants, there are no folds appearing in the epithelium until the formation of mitotic domains (about 10 min). Then, a large ectopic fold appears posterior to dividing cells (15 min). The epithelium of *eve* mutants shows additional folding events along the head and trunk regions. Frame rate = 10 fps. Scale bar = 20 µm.

## Supplementary Video 6


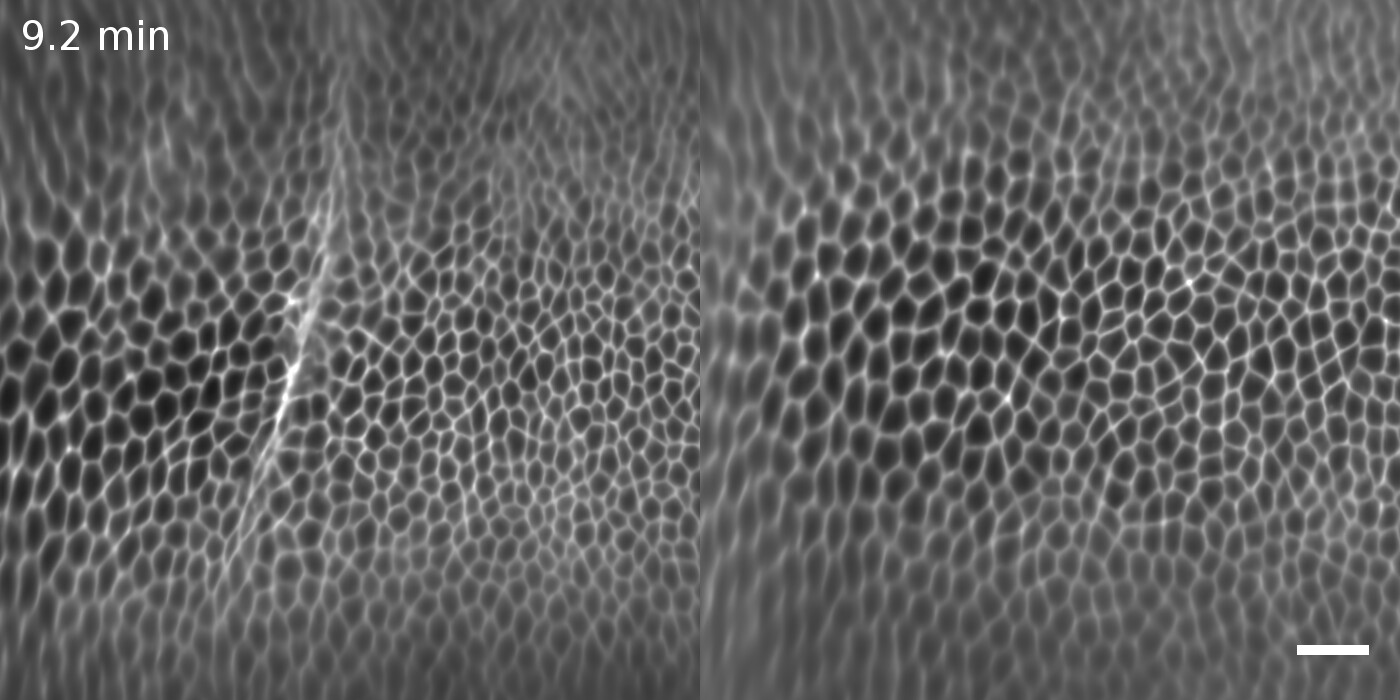


Supplementary Video 6: Ectopic folding between mitotic domains in *btd* mutant. Detailed view of cartographic projections of *btd* embryos, showing the formation of the cephalic furrow (left) and of an ectopic fold (right). In sibling controls, the cephalic furrow initiates from a narrow row of cells and invaginates in a progressive manner before the appearance of mitotic domains. In *btd* mutants, the ectopic folds only appear after the apical expansion of dividing cells within mitotic domains, quickly buckling and unfolding shortly after. Frame rate = 10 fps. Scale bar ≈ 20 µm.

## Supplementary Video 7


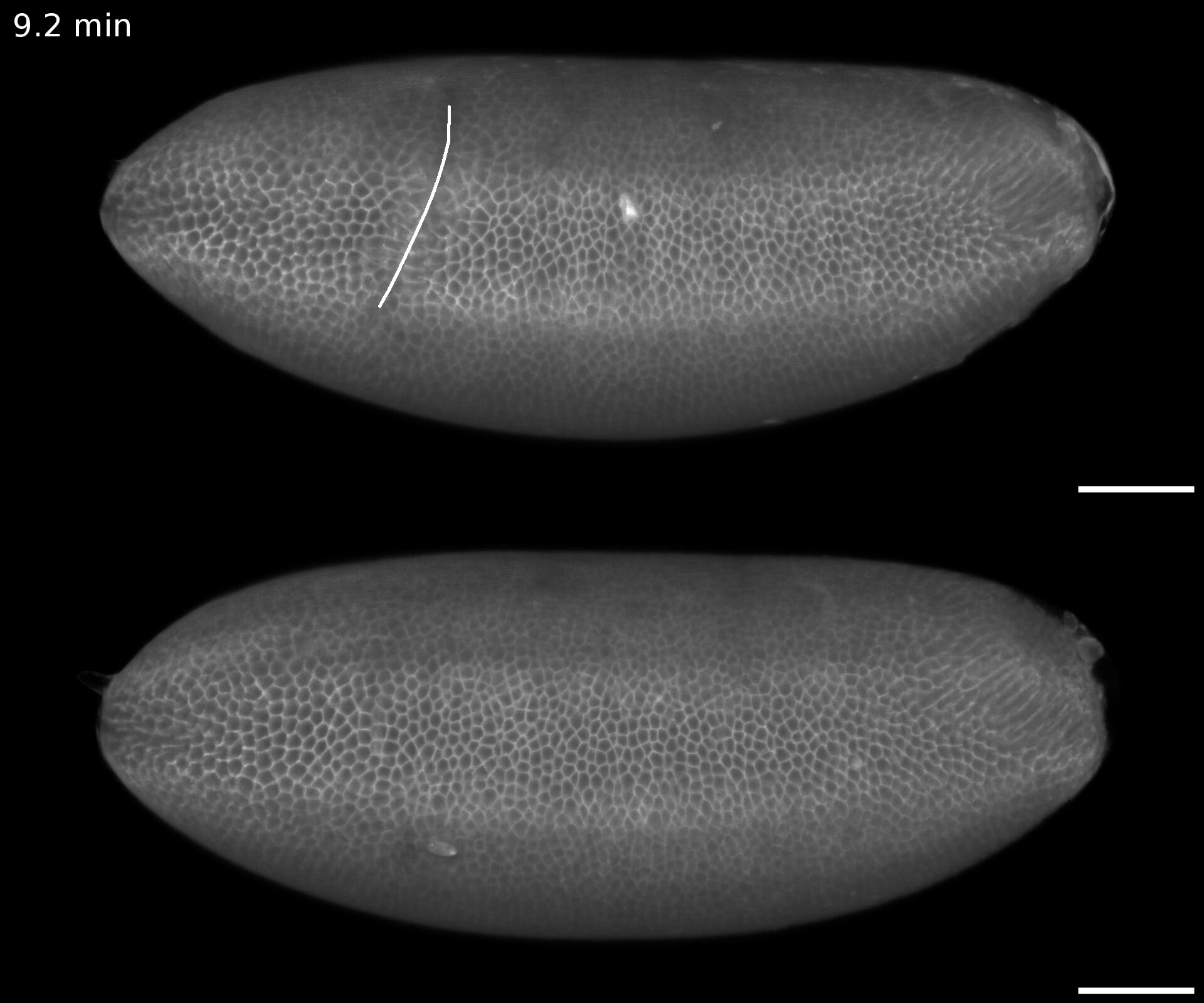


Supplementary Video 7: Dynamics of ectopic folding in *btd* mutant. The cephalic furrow in sibling controls (top) and the ectopic folds in *btd* mutants (bottom) are annotated in white to visualize the dynamics in position, extension, and shape during their formation. Frame rate = 10 fps. Scale bars = 50 µm.

## Supplementary Video 8


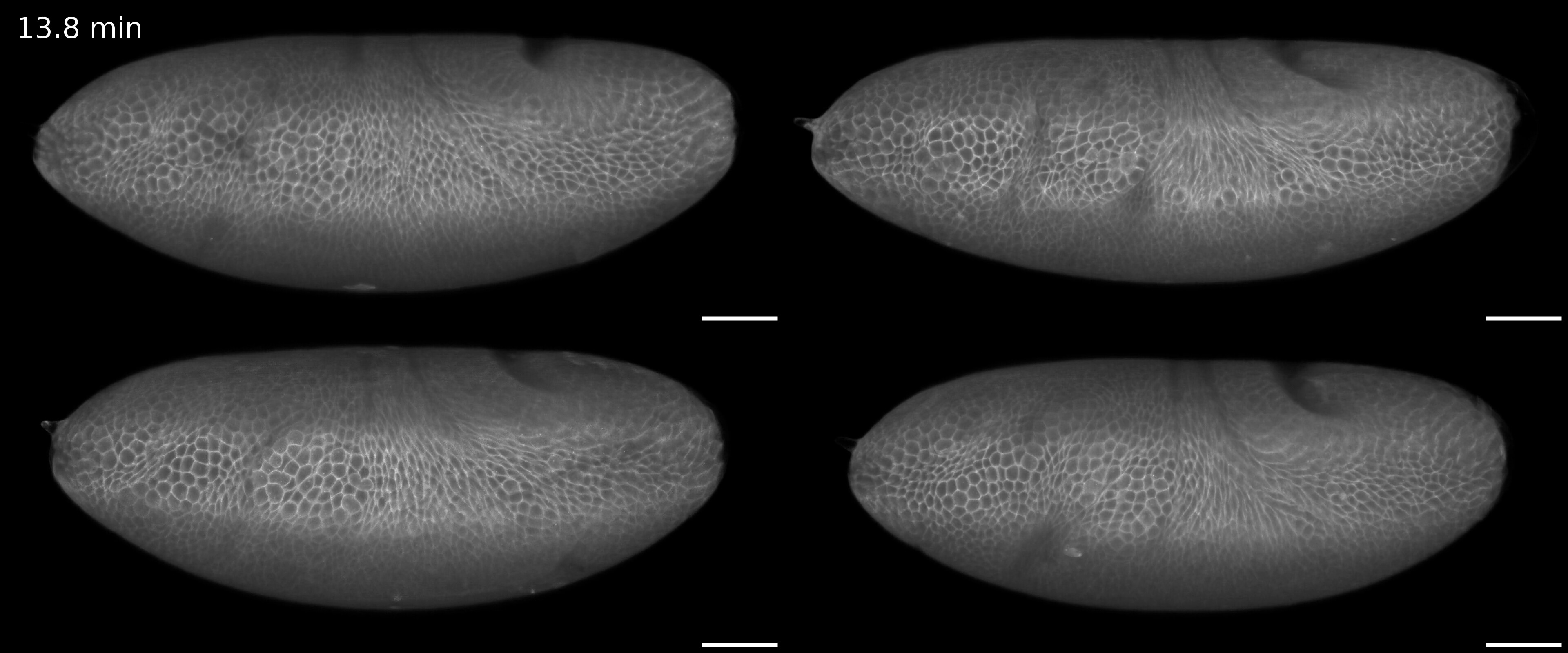


Supplementary Video 8: Variability of ectopic folding in *btd* mutants. The video shows four individual *btd* mutants, where each displays a different pattern and number of ectopic folds at the head–trunk interface. The video is looped to highlight the dynamics of ectopic folding. Frame rate = 15 fps. Scale bars = 50 µm.

## Supplementary Video 9


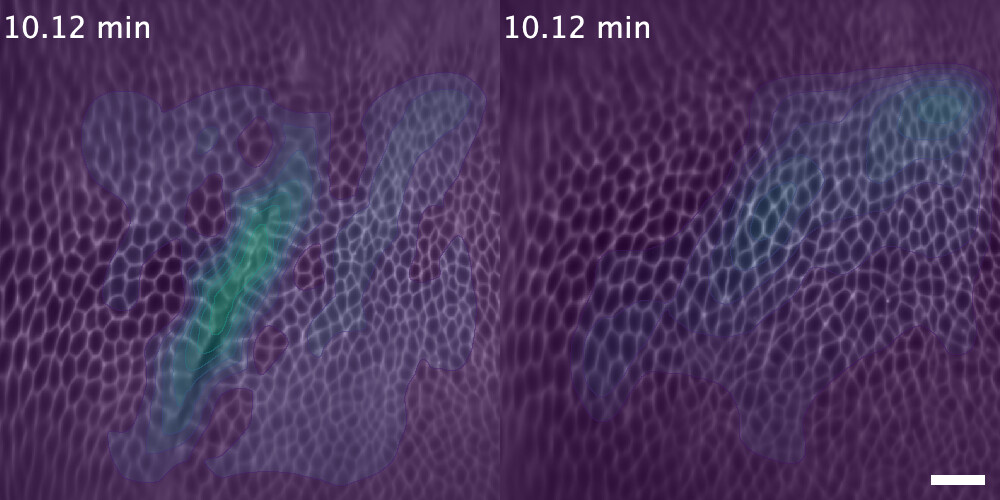


Supplementary Video 9: Epithelial strain rate during ectopic folding in *btd* mutant. Video from [Supplementary Video 6](#fig%3Avid-mitotic-fold) overlaid with the estimated strain rate across the tissues (color-coded from purple to yellow). Increases in strain rates are associated with tissue infolding and mitotic expansions. The video is looped. Frame rate = 10 fps. Scale bar ≈ 20 µm.

## Supplementary Video 10


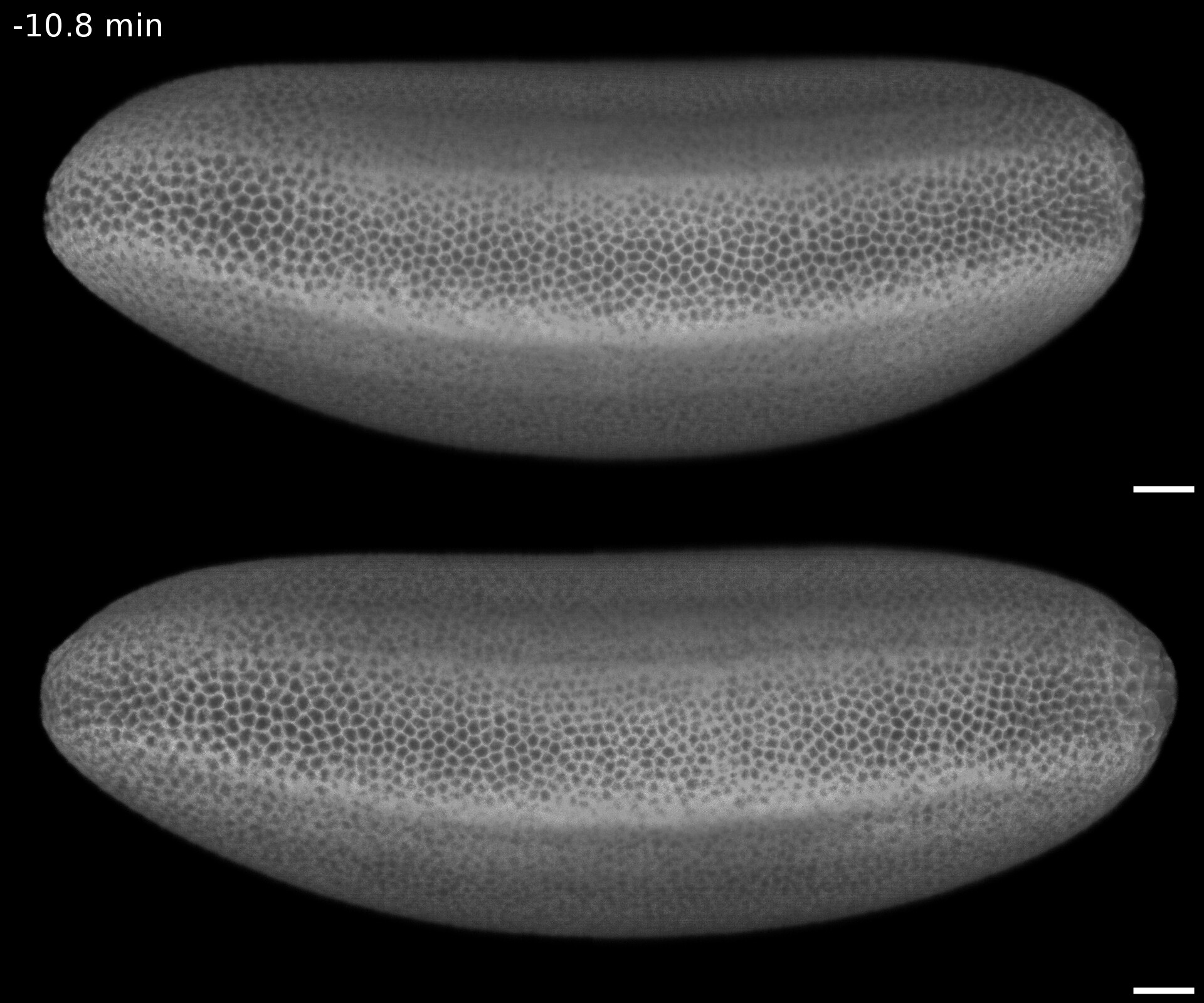


Supplementary Video 10: Lateral view of cephalic furrow formation in *stg* mutants. Sibling control (top) and *stg* mutant (bottom) during gastrulation. The formation of the cephalic furrow is almost identical to the control embryo. The other morphogenetic movements also occur normally until about 35 min. At this point, the cells in the *stg* mutant are notably larger than the control. Frame rate = 15 fps. Scale bars = 50 µm.

## Supplementary Video 11


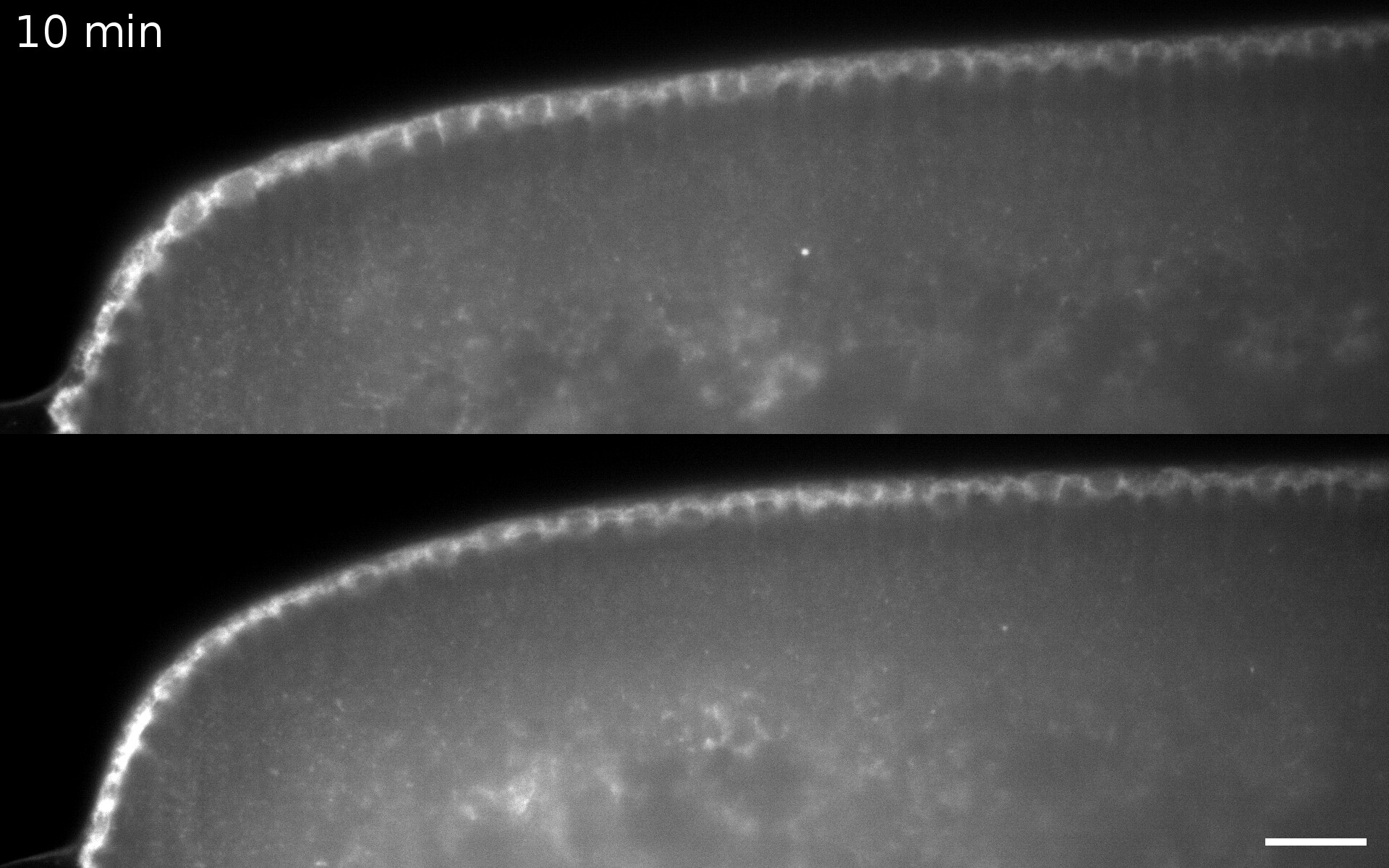


Supplementary Video 11: Dorsal view of cephalic furrow formation in *stg* mutants. Sibling control (top) and *stg* mutant (bottom) during gastrulation. The cephalic furrow in *stg* mutants initiates without delay and shows identical morphology to the control until cell divisions begin in the latter. The cells dividing within the cephalic furrow of control embryos alter its morphology; it becomes curved and lengthier. In contrast, the cephalic furrow in the *stg* mutant retains its initial morphology until it unfolds. Frame rate = 10 fps. Scale bar = 20 µm.

## Supplementary Video 12


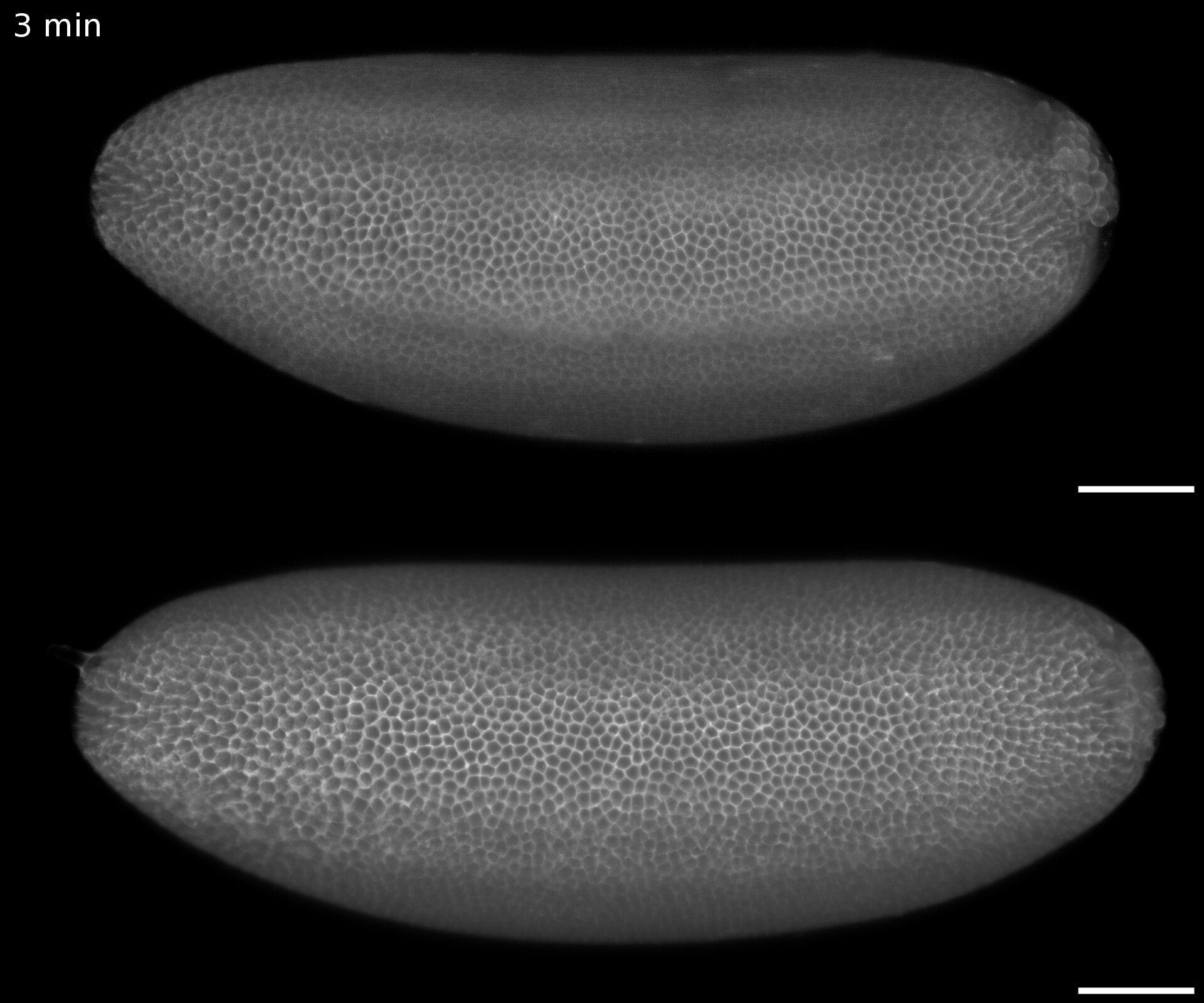


Supplementary Video 12: Lateral view of *btd–stg* double mutant. A *btd* homozygote (top) shows the formation of ectopic folds, while no ectopic folds form in the *btd–stg* double mutant (bottom). Frame rate = 10 fps. Scale bars = 50 µm.

## Supplementary Video 13


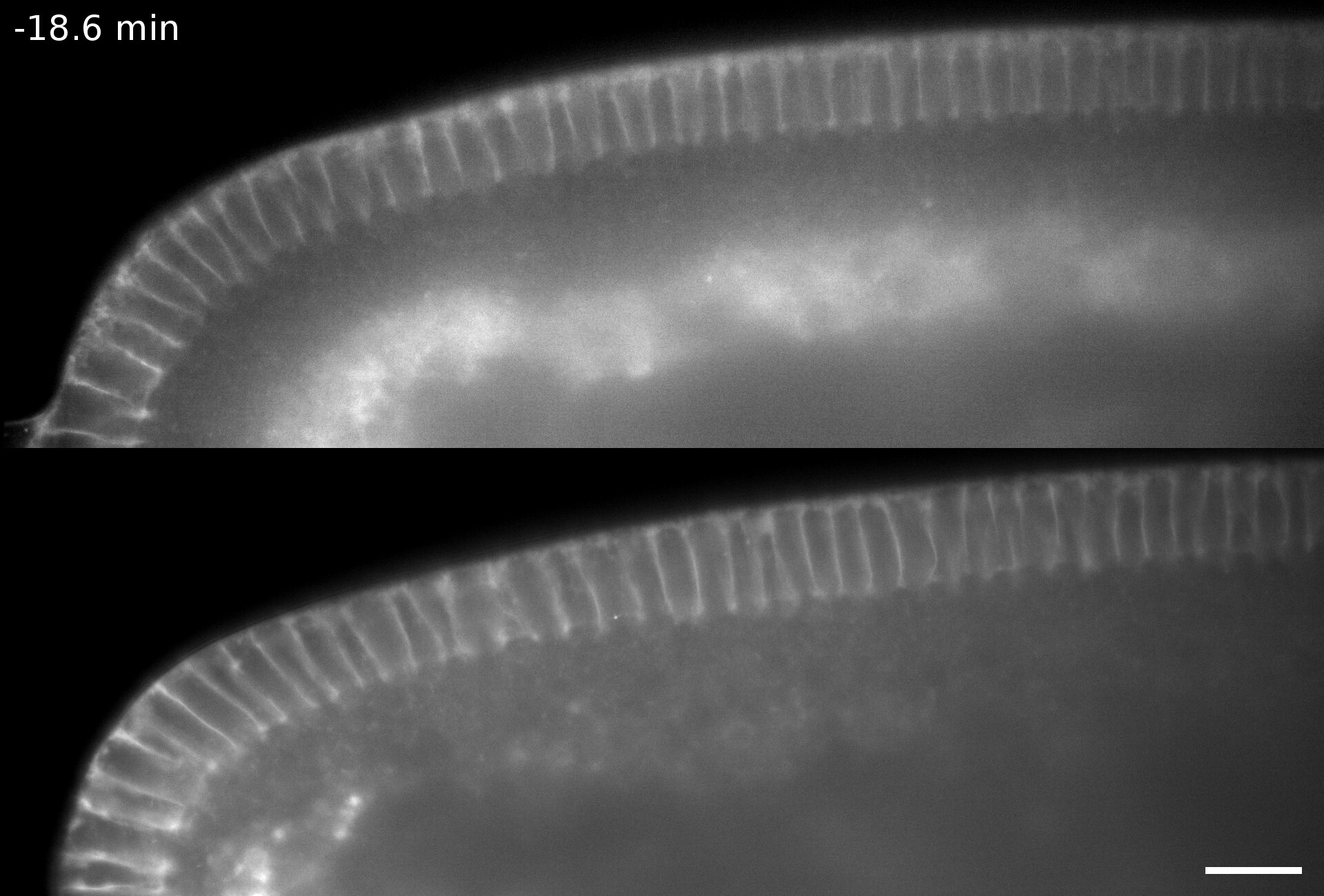


Supplementary Video 13: Dorsal view of *btd–stg* double mutant. A *btd* homozygote shows the formation of an ectopic fold (top). The *btd–stg* double mutant exhibits no mitotic domains and no ectopic folds (bottom). Frame rate = 10 fps. Scale bar = 20 µm.

## Supplementary Video 14


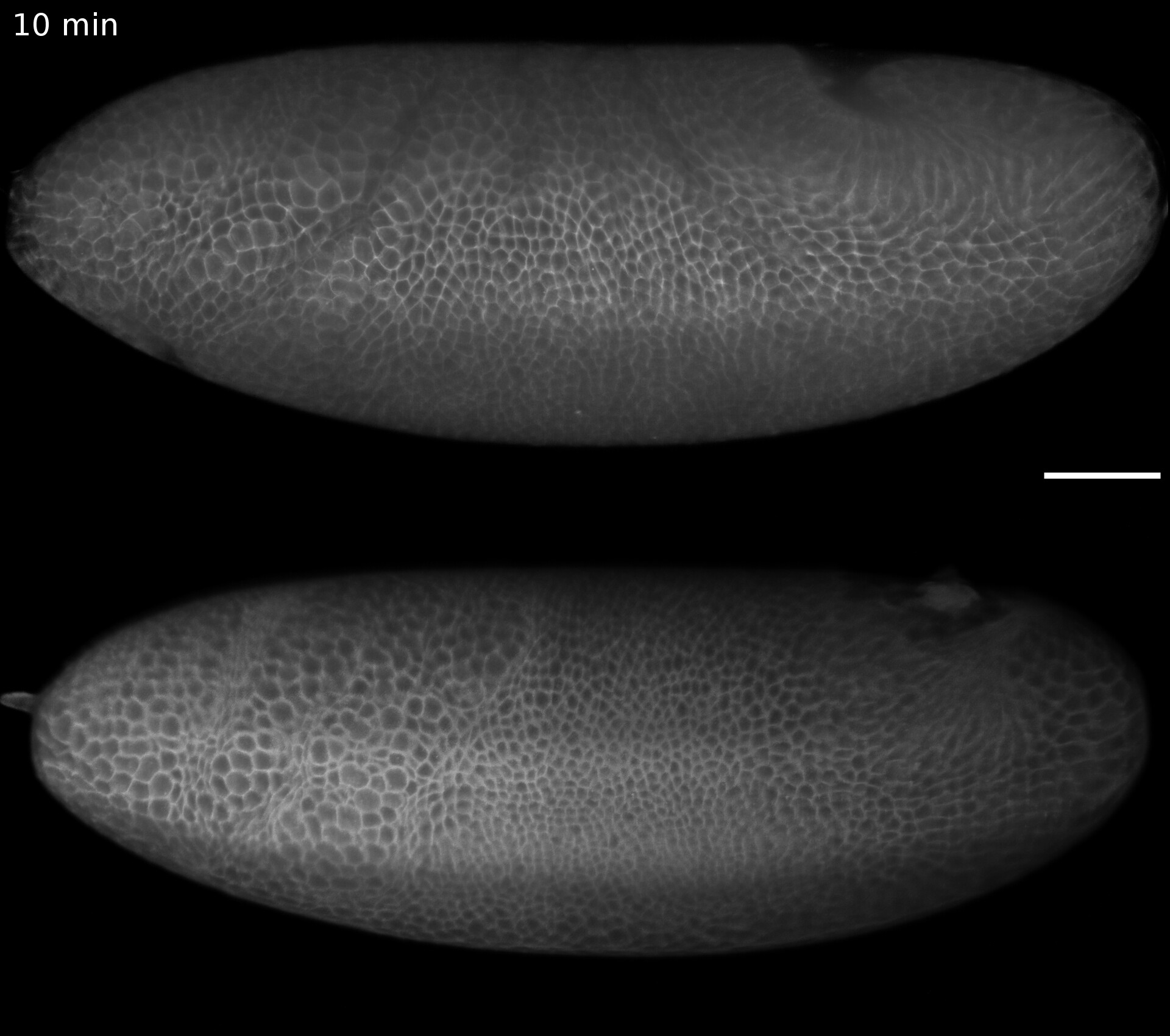


Supplementary Video 14: Lateral views of germ band cauterization in *eve* embryos. Non-cauterized *eve* embryo showing the formation of ectopic folds (top, same embryo from [Supplementary Video 3](#fig%3Avid-lateral-eve)) and a cauterized *eve* embryo where no ectopic folds appear at the head–trunk interface (bottom). The germ band extension is mechanically blocked by cauterizing the tissue to the vitelline envelope. Mitotic domains form normally, but no folding of the surface occurs. Frame rate = 10 fps. Scale bar = 50 µm.

## Supplementary Video 15


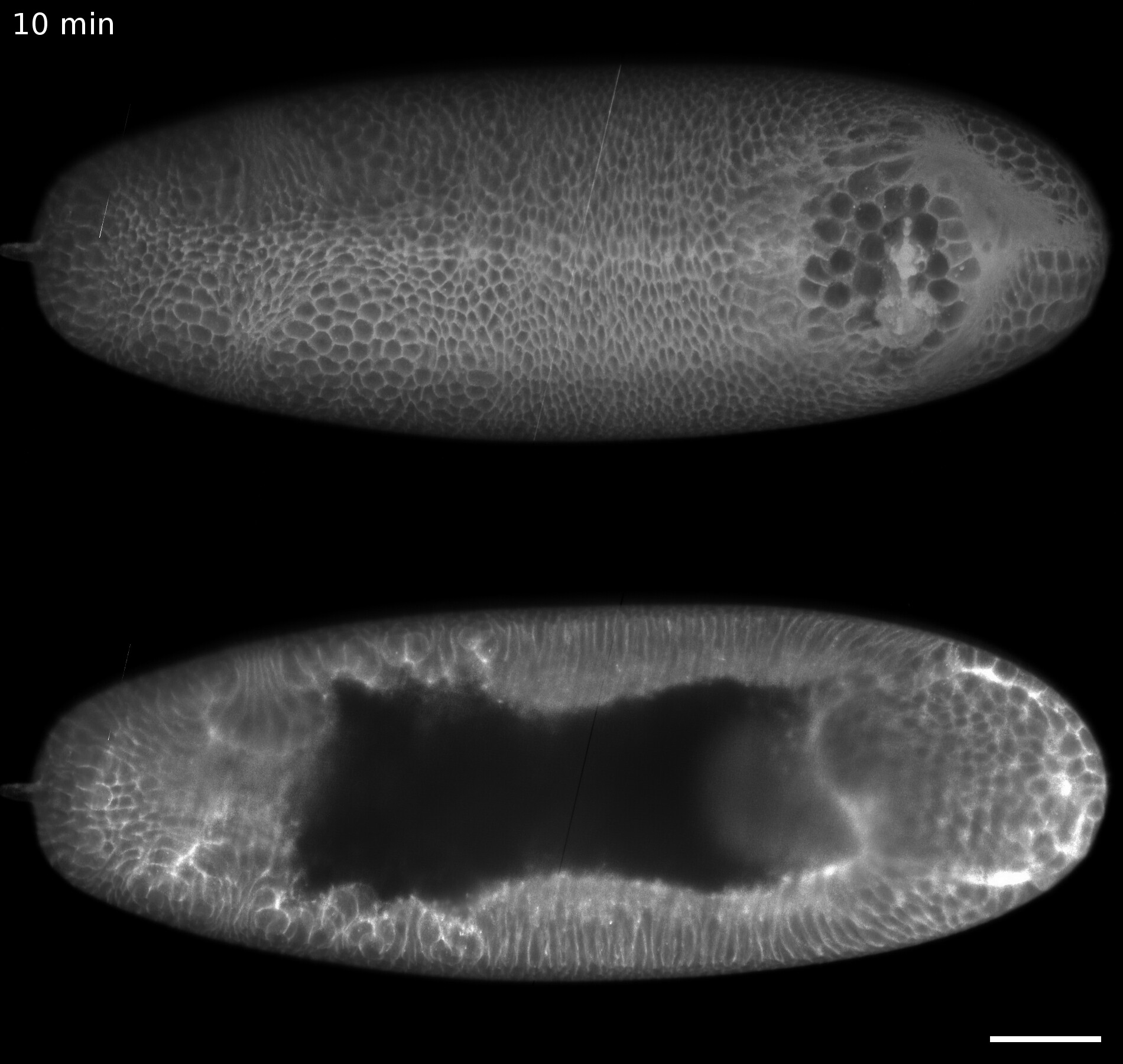


Supplementary Video 15: Profile views of germ band cauterization in *eve* mutant. Same embryo from [Supplementary Video 14](#fig%3Avid-cautlat-eve), but showing a surface and a profile view. The cauterization prevents the extension of the germ band. The mitotic domains compress non-dividing cells, but these do not buckle. Frame rate = 10 fps. Scale bar = 50 µm.

## Supplementary Video 16


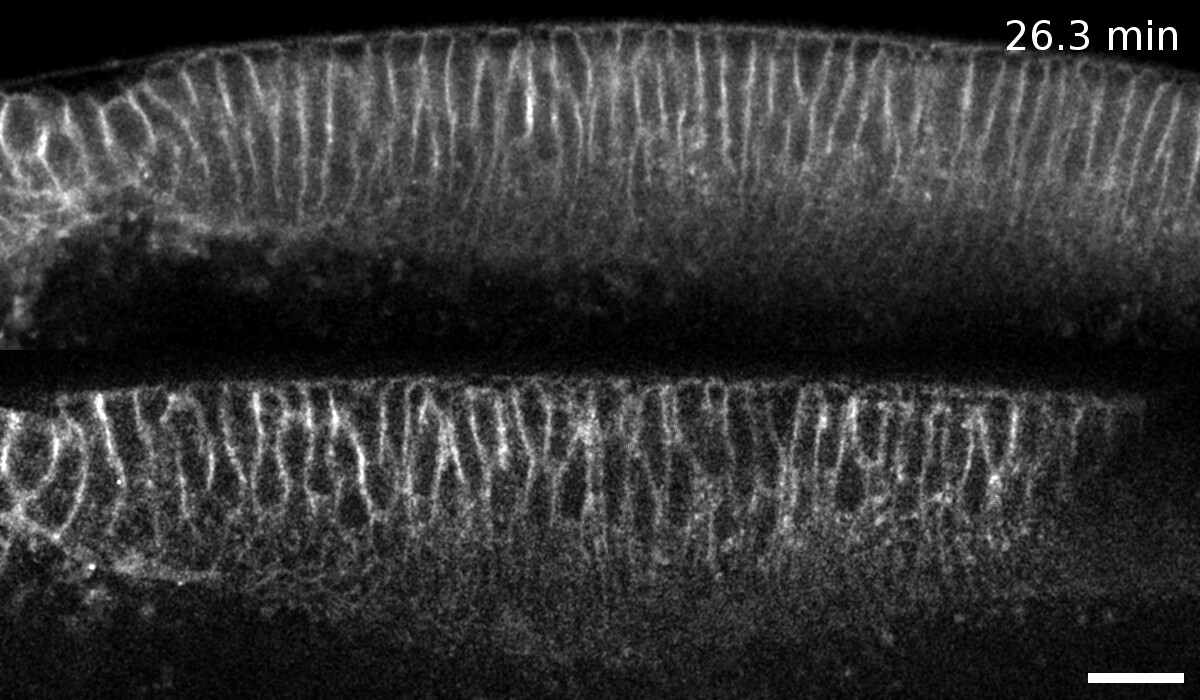


Supplementary Video 16: Profile views of germ band cauterizations in *btd* embryos. A non-cauterized *btd* embryo (top) showing ectopic folds and a cauterized *btd* embryo showing no ectopic folds (bottom). Frame rate = 10 fps. Scale bar = 20 µm.

## Supplementary Video 17


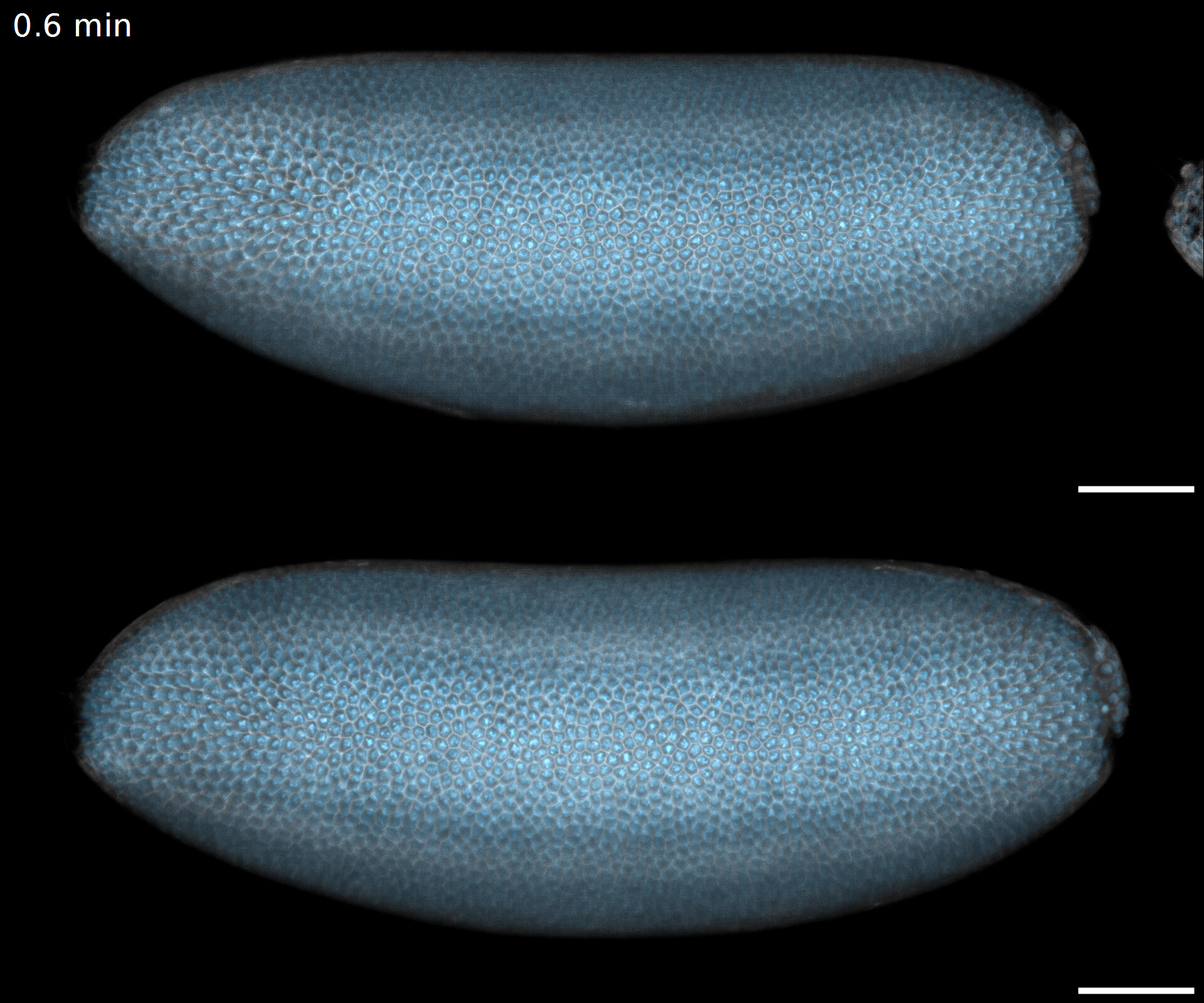


Supplementary Video 17: Lateral view of *slp* mutant. A *slp* heterozygote (top) shows a normal cephalic furrow formation. The *slp* homozygote (bottom) shows a delayed initiator cell behavior and cephalic furrow formation. The embryo also exhibits a more prominent posterior dorsal fold and an ectopic fold forming within the mitotic domain 6 before cell divisions. Frame rate = 10 fps. Scale bar = 50 µm.

## Supplementary Note 1

### Disruption of initiator cell behavior in cephalic furrow mutants

We analyzed how the behavior of initiator cells is perturbed in the *btd*, *eve*, and *prd* mutant backgrounds using lightsheet microscopy. In wildtype embryos, the initiator cells shorten and undergo anisotropic apical constriction minutes before the end of cellularization[^7^](#ref-Spencer2015-td) ([Extended Data Fig. 1](#fig%3Ainitiator)a,b). In *prd* mutants, both behaviors are delayed in initiator cells, and the adjacent cells lack the typical arched profile present in the wildtype invagination[^7^](#ref-Spencer2015-td) ([Extended Data Fig. 1](#fig%3Ainitiator)a,b). Moreover, the tissue only invaginates after gastrulation and the resulting fold is abnormal compared to a wildtype invagination. In *btd* and *eve* mutants, the cephalic furrow phenotypes are stronger. Initiator cells in *btd* embryos exhibit a reduced degree of apical constriction but do not shorten ([Extended Data Fig. 1](#fig%3Ainitiator)a,b, [Supplementary Video 1](#fig%3Avid-initiator)). In contrast, *eve* mutants show neither apical constriction nor cell shortening; the epithelium remains flat for several minutes after gastrulation ([Extended Data Fig. 1](#fig%3Ainitiator)a,b). These observations reveal that initiator cell behavior in *prd* mutants is not only delayed but also perturbed and that the cellular mechanism that drives cephalic furrow formation is severely disrupted in *btd* and *eve* mutants.

## Supplementary Note 2

### Design of physical model and simulations

Our model represents an epithelial monolayer confined inside a rigid shell. It embodies one side of a frontal slice between the midline and the dorsal apex of a *Drosophila* embryo with its typical morphological proportions (Fig. [3](#fig%3Afold-model)a, [Extended Data Fig. 4](#fig%3Amodel-features)a). The blastoderm is modeled by an elliptical arc of equidistant particles connected by springs and enclosed on one side by a rigid barrier representing the vitelline envelope (Fig. [3](#fig%3Afold-model)b). The total energy per unit length of this tissue ($W_{T}$) is a sum of a stretching energy component ($W_{s}$) and a bending energy component ($W_{b}$) (Fig. [3](#fig%3Afold-model)c). Each of these components has a rigidity associated with them. $K_{s}$ is the stretching rigidity and $K_{b}$ is the bending rigidity. These two parameters can be combined into a single dimensionless bending rigidity, $K_{b}^{\text{*}}$ (Fig. [3](#fig%3Afold-model)c).

To simulate the physical interactions between mitotic domains, germ band, and cephalic furrow, we defined the mitotic domains as compressed regions that tend to expand (they contain more particles compared to the surrounding regions) and the cephalic furrow as a narrow region having an intrinsic negative curvature predisposing the tissue to invaginate (Fig. [3](#fig%3Afold-model)b). The germ band is defined by the position of the posterior end of the tissue ($g$), which is fixed at different fractions of egg length for each simulation (Fig. [3](#fig%3Afold-model)a,b). Thus, the effect of germ band extension appears as a global compression in the blastoderm. To run the simulations, we defined a ground level of random noise and iterated towards equilibrium of the total energy in the system (see Methods). We then set several simulations with different bending rigidity values and combinations of presence/absence of mitotic domains, cephalic furrow, and percentages of germ band extension to quantify the position, frequency, and depth of ectopic folding in the epithelium.

To characterize the model properties and energy dynamics, we ran simulations using initially a single bending rigidity value, without mitotic domains, and at different percentages of germ band extension. Without the germ band, the tissue is almost stress-free, and no ectopic folding occurs (Fig. [3](#fig%3Afold-model)e). We begin to observe folds in the simulations with higher progression of germ band extension. Folding events are stochastic and happen at distinct iterations for each simulation. When a fold begins to form, the bending energy increases, releasing a larger amount of stretching energy, which, in turn, decreases the total energy of the system over each iteration (Fig. [3](#fig%3Afold-model)d). The increase in bending energy coincides with a rapid deepening of the fold. Once the bending energy reaches a peak, we find that the fold continues to deepen more gradually, but the number of folds rarely changes afterward (Fig. [3](#fig%3Afold-model)d, [Extended Data Fig. 4](#fig%3Amodel-features)b). Therefore, this peak of bending energy provides an informative reference point, which we used to standardize the comparison across simulations.

## Supplementary Note 3

### Comparison of reference bending rigidity to direct measurements

To compare the reference bending rigidity that we established for the *Drosophila* blastoderm based on our experimental data ($K_{b}^{\text{*}}\approx1.0\times{10}^{-4}$) with direct measurements of the bending rigidity, we calculated the $K_{b}^{\text{*}}$ of 3D-cultured epithelial monolayers described in the literature. Trushko et al.[^17^](#ref-Trushko2020-gf) reports that in a MDCK monolayer $K_{b}=5\times{10}^{-13}Nm$ and $K_{s}=0.15Nm^{-1}$. With these values, we can compute the dimensionless bending rigidity $K_{b}^{\text{*}}$. However, the thickness of the MDCK monolayer (20 µm) and the *Drosophila* blastoderm (40 µm) are different, and hence we need to correct for this before computing $K_{b}^{\text{*}}$ for the MDCK monolayer. Given that, in elastic sheets, the bending rigidity scales with the square of the thickness of the tissue (${K_{b}}/{K_{s}}\propto h^{2}$),[^64^](#ref-Efrati2009-ve) we can compute the corrected $K_{b}$ as $K_{b}^{'}=K_{b}^{\text{*}}({h_{blastoderm}}/{h_{MDCK}})^{2}$. This gives us $K_{b}^{'}=2\times{10}^{-12}Nm$. Now, we can calculate $K_{b}^{\text{*}}={K_{b}^{'}}/{K_{s}}L^{2}$ by adjusting ${K_{b}^{'}}/{K_{s}}$ to the relevant length scale, the semi-major axis of the embryo. As the average *Drosophila* egg length is 510 µm,[^65^](#ref-Markow2009-xc) we used $L=255\mu m$. The final value we obtain is $K_{b}^{\text{*}}=2.05\times{10}^{-4}$. This is the estimated $K_{b}^{\text{*}}$ for the MDCK monolayer with the same thickness (height-corrected) and geometrical conditions (length-corrected) as the *Drosophila* blastoderm.

## Supplementary Note 4

### Live-imaging screen for cephalic furrow genes

To uncover other genes directly involved in cephalic furrow formation in addition to *btd*, *eve*, and *prd*, we performed a live-imaging screen in strains containing loss-of-function alleles for a selection of candidate genes expressed at the head–trunk region.[^66^](#ref-Tomancak2002-yf)^–^[^68^](#ref-Lecuyer2007-uy) Because the cephalic furrow is transient and leaves no trace, the live-imaging approach is critical to recognize altered phenotypes. From about 50 genes, we only detected three showing abnormal cephalic furrow formation to different degrees besides the previously described genes (see [Supplementary Table 1](#tbl%3Ascreen)). The strongest cephalic furrow phenotype was present in flies mutant for the *sloppy paired* (*slp*) genes.

## Supplementary Note 5

### Role of sloppy paired in cephalic furrow formation

To identify other cephalic furrow genes, we performed a live-imaging screen using loss-of-function alleles of several candidates expressed at the head–trunk interface (Supplementary Note 4). We found that null mutants for the *sloppy paired* (*slp*) transcription factors, *slp1* and *slp2*, show a strong phenotype where the cephalic furrow is delayed and shifted towards the anterior end by ~6% of the egg length (control=67.6±1.4%, n=26; *slp*=73.2±0.7%, n=7) ([Extended Data Fig. 5](#fig%3Aslp-analyses)a,b, [Supplementary Video 17](#fig%3Avid-slp-lateral)). With this anterior shift, *slp* mutants exhibit a more prominent posterior dorsal fold and an early ectopic fold within MD6 appearing before cell divisions ([Extended Data Fig. 5](#fig%3Aslp-analyses)a, [Supplementary Video 17](#fig%3Avid-slp-lateral)). These observations are congruent with the increase in posterior mechanical instability present in our simulations where the cephalic furrow is shifted forward (Fig. [3](#fig%3Afold-model)h).

Since *slp1* is a known anterior repressor that positions anterior pair-rule stripes,[^18^](#ref-Andrioli2012-fv)^,^[^69^](#ref-Andrioli2004-qi) we wondered if the shift in the position of the cephalic furrow in *slp* mutants coincided with a shift in typical overlap between *btd* and *eve* stripe 1 at the head–trunk boundary. We find that the expression patterns and typical overlap between the two genes remain almost unaltered, except for the small ectopic expression of *btd* in the head and for the wider gap between *eve* stripes 1 and 2 ([Extended Data Fig. 5](#fig%3Aslp-analyses)c–e). The anterior shift in cells expressing *btd* and *eve* corresponds to a few rows of blastoderm cells (control=4.7±0.5 rows, n=4; *slp*=7.3±0.7 rows, n=5) ([Extended Data Fig. 5](#fig%3Aslp-analyses)f). Despite the displacement, most *slp* embryos exhibit initiator cell behaviors, suggesting that the patterning is not entirely perturbed ([Extended Data Fig. 5](#fig%3Aslp-analyses)g). However, the symmetry of the resulting fold is altered ([Extended Data Fig. 5](#fig%3Aslp-analyses)h), indicating that *slp1* may contribute not only to the positioning but also to the patterning of individual cells that give rise to the cephalic furrow.

## Supplementary Note 6

### Genetic interactions at the head–trunk boundary

To better understand the genetic interactions at the head–trunk boundary of *Drosophila*, we analyzed the expression of *btd*, *eve*, *prd*, and *slp1* transcripts in cephalic furrow mutants ([Extended Data Fig. 7](#fig%3Amutant-expression)). Our analysis of *slp* mutants reveals that its early activity represses *eve* expression at the anterior end and ultimately determines the position of the head–trunk *btd* domain and *eve* stripe 1 without disrupting the *btd–eve* overlap ([Extended Data Fig. 5](#fig%3Aslp-analyses), Supplementary Note 5). In *btd* mutants, we observe *slp1* transcripts between the *slp1* anterior head domain and the *slp1* stripe 1 ([Extended Data Fig. 7](#fig%3Amutant-expression)a,b), suggesting that *btd* may repress *slp1* in this region in wildtype embryos. The expression of *eve* stripe 1 is diminished, as previously reported,[^8^](#ref-Vincent1997-fa) and the expression of *prd* stripe 1 is disrupted in *btd* mutants. This indicates that *btd* activity may be important to activate or maintain stripe 1 expression of *eve* and *prd*. These changes disrupt the typical molecular arrangement of *slp1*, *eve*, and *prd* in the head–trunk epithelium, which could explain the faulty initiator cell behavior in *btd* mutants. Moreover, in *btd* mutants, *eve* stripe 2 and the adjacent *slp1* and *prd* stripes are shifted forward and the head–trunk domain of *btd* itself is expanded posteriorly. Therefore, while *slp* activity positions stripe 1 upstream, *btd* activity controls the positioning of stripe 2 further downstream. In *eve* mutants, *slp1* and *prd* expression at the head–trunk boundary are de-repressed, but to a lesser degree than in the trunk, where the transcripts become ubiquitous ([Extended Data Fig. 7](#fig%3Amutant-expression)a,b,c). The expression of *eve* stripe 1 is weaker. Unlike *btd* mutants, the positioning of domains is not affected in *eve* mutants. In *prd* mutants, *slp1* stripes 1 and 2 are fused, and *eve* stripes are wider and less sharp compared to wildtype embryos ([Extended Data Fig. 7](#fig%3Amutant-expression)a,b,c). This suggests that *prd* activity is important to sharpen the expression domains around the initiator cells to single-row resolution during the patterning of the cephalic furrow.

## Supplementary Note 7

### Role of tissue compression and cell divisions in buckling

Mechanical compression is a key mechanism that drives the formation of epithelial folds in embryonic development,[^3^](#ref-Collinet2021-qa)^,^[^70^](#ref-Nelson2016-gz) particularly when tissues are under confinement.[^17^](#ref-Trushko2020-gf) The occurrence of cell divisions in epithelial monolayers can create instability in the tissue. This occurs due to the in-plane, outward forces generated during the elongation phase of mitosis.[^71^](#ref-Gupta2021-oo) Moreover, the basal portion of the cell can detach during division and cause an imbalance in the epithelium.[^72^](#ref-Ko2020-wk) This process occurs in the tracheal placode of flies[^24^](#ref-Kondo2013-gy) and intestinal villi of mice,[^25^](#ref-Freddo2016-et) where the mitotic rounding of dividing cells induces the formation of epithelial folds. Interestingly, these studies found that folding only occurs when the epithelium is under compression. This is similar to our findings that only the combined action of mitotic expansions and germ band extension can induce ectopic folds in the *Drosophila* blastoderm. These observations reveal a common mechanism by which epithelial folds can form during embryonic development.

## Supplementary Table 1

Table Supplementary Table 1: Summary of the results of the live-imaging screen for cephalic furrow genes.

| gene name | gene symbol | allele/deficiency | phenotype |
| --- | --- | --- | --- |
| *buttonhead* | *btd* | *btd*^XA^ | absent[^8^](#ref-Vincent1997-fa) |
| *even skipped* | *eve* | *eve*^3^ | absent[^8^](#ref-Vincent1997-fa) |
| *paired* | *prd* | *prd*^4^ | delayed[^15^](#ref-Blankenship2001-tm) and abnormal |
| *sloppy paired 1* and *2* | *slp1*, *slp2* | *slp*^Δ34B^ | delayed and displaced anteriorly |
| *sloppy paired 1* | *slp1* | *slp1*^1^ | delayed and displaced anteriorly |
| *giant* | *gt* | *gt*^X11^ | perturbed formation |
| *knirps*, *knirps-like* | *kni*, *knrl* | Df(3L)BSC448 | ventral portion displaced anteriorly |

64. Efrati, E., Sharon, E. & Kupferman, R. Elastic theory of unconstrained non-euclidean plates. *J. Mech. Phys. Solids* **57,** 762–775 (2009).

65. Markow, T. A., Beall, S. & Matzkin, L. M. Egg size, embryonic development time and ovoviviparity in *Drosophila* species. *J. Evol. Biol.* **22,** 430–434 (2009).

66. Tomancak, P., Beaton, A., Weiszmann, R., Kwan, E., Shu, S., Lewis, S. E., Richards, S., Ashburner, M., Hartenstein, V., Celniker, S. E. & Rubin, G. M. Systematic determination of patterns of gene expression during *Drosophila* embryogenesis. *Genome Biol.* **3,** research0088.1 (2002).

67. Tomancak, P., Berman, B. P., Beaton, A., Weiszmann, R., Kwan, E., Hartenstein, V., Celniker, S. E. & Rubin, G. M. Global analysis of patterns of gene expression during *Drosophila* embryogenesis. *Genome Biol.* **8,** R145 (2007).

68. Lécuyer, E., Yoshida, H., Parthasarathy, N., Alm, C., Babak, T., Cerovina, T., Hughes, T. R., Tomancak, P. & Krause, H. M. Global analysis of mRNA localization reveals a prominent role in organizing cellular architecture and function. *Cell* **131,** 174–187 (2007).

69. Andrioli, L. P., Oberstein, A. L., Corado, M. S. G., Yu, D. & Small, S. Groucho-dependent repression by Sloppy-paired 1 differentially positions anterior pair-rule stripes in the *Drosophila* embryo. *Dev. Biol.* **276,** 541–551 (2004).

70. Nelson, C. M. On buckling morphogenesis. *J. Biomech. Eng.* **138,** 021005 (2016).

71. Gupta, V. K., Nam, S., Yim, D., Camuglia, J., Martin, J. L., Sanders, E. N., O’Brien, L. E., Martin, A. C., Kim, T. & Chaudhuri, O. The nature of cell division forces in epithelial monolayers. *J. Cell Biol.* **220,** (2021).

72. Ko, C. S., Kalakuntla, P. & Martin, A. C. Apical constriction reversal upon mitotic entry underlies different morphogenetic outcomes of cell division. *Mol. Biol. Cell* **31,** 1663–1674 (2020).
