# Supplementary material for: Patterned invagination prevents mechanical instability during gastrulation
Source: Nature. 2025 Sep 3;646(8085):627–36. doi: 10.1038/s41586-025-09480-3 (PMC12527948; doi:10.1038/s41586-025-09480-3)
Supplement: Supplementary file 2 — Reporting Summary [file 41586_2025_9480_MOESM2_ESM.pdf]

Corresponding author(s): Pavel Tomancak, Bruno Vellutini

Last updated by author(s): Jul 11, 2025

Reporting Summary

Nature Portfolio wishes to improve the reproducibility of the work that we publish. This form provides structure for consistency and transparency in reporting. For further information on Nature Portfolio policies, see our Editorial Policies and the Editorial Policy Checklist.

Statistics

For all statistical analyses, confirm that the following items are present in the figure legend, table legend, main text, or Methods section.

n/a	Confirmed	
☐	☒	The exact sample size (*n*) for each experimental group/condition, given as a discrete number and unit of measurement
☐	☒	A statement on whether measurements were taken from distinct samples or whether the same sample was measured repeatedly
☐	☒	The statistical test(s) used AND whether they are one- or two-sided<br>*Only common tests should be described solely by name; describe more complex techniques in the Methods section.*
☒	☐	A description of all covariates tested
☒	☐	A description of any assumptions or corrections, such as tests of normality and adjustment for multiple comparisons
☐	☒	A full description of the statistical parameters including central tendency (e.g. means) or other basic estimates (e.g. regression coefficient) AND variation (e.g. standard deviation) or associated estimates of uncertainty (e.g. confidence intervals)
☐	☒	For null hypothesis testing, the test statistic (e.g. *F*, *t*, *r*) with confidence intervals, effect sizes, degrees of freedom and *P* value noted<br>*Give P values as exact values whenever suitable.*
☒	☐	For Bayesian analysis, information on the choice of priors and Markov chain Monte Carlo settings
☒	☐	For hierarchical and complex designs, identification of the appropriate level for tests and full reporting of outcomes
☒	☐	Estimates of effect sizes (e.g. Cohen's *d*, Pearson's *r*), indicating how they were calculated

Our web collection on statistics for biologists contains articles on many of the points above.

Software and code

Policy information about availability of computer code

Data collection	We acquired our live imaging data using a Zeiss Lightsheet Z.1 microscope running ZEN 2014 SP1 v9.2.10.54. For imaging fixed samples from in situ hybridization experiments, we used an inverted Zeiss LSM 700 confocal microscope running ZEN 2012 SP5 FP3 v14.0.25. We performed laser cauterization experiments in two microscope setups, a lightsheet Luxendo MuVi SPIM with a photomanipulation module and a confocal Zeiss LSM 780 NLO with multiphoton excitation running ZEN Black v14.024.201. For the laser ablation experiments, we used a Yokogawa CSU-X1 spinning disk confocal microscope running AndorIQ for acquisition and an inverted Zeiss Axio Observer.Z1 spinning disk confocal microscope running ZEN Blue v3.2 with a Rapp OptoElectronic setup for photo-manipulation running SysCon2. All the data supporting the findings of this study are available on the Zenodo repositories with the identifiers https://doi.org/10.5281/zenodo.7781947 (imaging data), https://doi.org/10.5281/zenodo.7784906 (simulation data), and https://doi.org/10.5281/zenodo.7781916 (high-resolution figures and videos).
Data analysis	We processed imaging datasets and performed most image analyses using custom macros and plugins in Fiji/ImageJ v2.16.0/1.54p; Java 1.8.0_172. To improve the signal-to-noise ratio and Z-resolution of lateral lightsheet datasets, we used CARE CSBDeep v0.3.0. To generate 3D renderings, we used the Fiji plugin 3Dscript v0.2.1. We created cartographic projections using the ImSAnE toolbox v3a7be24 pipeline using MATLAB R2015b and ilastik v1.3.3b2. We used the Fiji plugin bUnwarpJ v2.6.13 to register the surface of individual embryos. We generated color-coded temporal projections using the script Temporal Color Code v101122 with the mpl-viridis color map, both bundled in Fiji. We used the Fiji plugin MorphoLibJ v1.6.0 to segment, measure, and color-code the cell apical areas, and the plugin Linear Stack Alignment with SIFT v1.5.0 to register cells between timepoints. We extracted measurements from the epithelium outlines using the Fiji plugin Analyze Skeleton v3.4.2. We generated kymographs using the Multi Kymograph v3.0.1 Fiji plugin. We created illustrations and assembled figure plates using Inkscape v1.2.2. For microscopy videos, we exported the original timelapse stacks to uncompressed AVI using Fiji and converted them to MPEG-4 format using HandBrake v1.6.1. We performed the data wrangling, statistical analyses, and plotting in R v4.2.1 using R Markdown notebooks in RStudio v2022.7.2.576, and in Python 3.10.7 using Jupyter notebooks v6.5.4. All the code necessary to reproduce the data

processing and downstream analyses of this study are available on the Zenodo repositories with the identifiers https://doi.org/10.5281/zenodo.7781947 (imaging analyses) and https://doi.org/10.5281/zenodo.7784906 (model analyses).

For manuscripts utilizing custom algorithms or software that are central to the research but not yet described in published literature, software must be made available to editors and reviewers. We strongly encourage code deposition in a community repository (e.g. GitHub). See the Nature Portfolio guidelines for submitting code & software for further information.

Data

Policy information about availability of data

All manuscripts must include a data availability statement. This statement should provide the following information, where applicable:

- Accession codes, unique identifiers, or web links for publicly available datasets
- A description of any restrictions on data availability
- For clinical datasets or third party data, please ensure that the statement adheres to our policy

All the data supporting the findings of this study have been deposited on Zenodo. The main repository containing the raw data, analyses pipelines, and source files for figures and text is available under the identifier https://doi.org/10.5281/zenodo.7781947. The imaging data for the lightsheet and in situ hybridization experiments is available under the identifier https://doi.org/10.5281/zenodo.15876638. The imaging data for the laser perturbation experiments is available under the identifier https://doi.org/10.5281/zenodo.15876646. The theory repository containing the code and scripts of the model, the output data of simulations, and notebooks of analyses and plotting is available under the identifier https://doi.org/10.5281/zenodo.7784906. The media repository containing the high-resolution figures and videos is available under the identifier https://doi.org/10.5281/zenodo.7781916.

Research involving human participants, their data, or biological material

Policy information about studies with human participants or human data. See also policy information about sex, gender (identity/presentation), and sexual orientation and race, ethnicity and racism.

Reporting on sex and gender	NA
Reporting on race, ethnicity, or other socially relevant groupings	NA
Population characteristics	NA
Recruitment	NA
Ethics oversight	NA

Note that full information on the approval of the study protocol must also be provided in the manuscript.

Field-specific reporting

Please select the one below that is the best fit for your research. If you are not sure, read the appropriate sections before making your selection.

☒ Life sciences ☐ Behavioural & social sciences ☐ Ecological, evolutionary & environmental sciences

For a reference copy of the document with all sections, see nature.com/documents/nr-reporting-summary-flat.pdf

Life sciences study design

All studies must disclose on these points even when the disclosure is negative.

Sample size	We performed no prior estimation of sample sizes for experiments. Following previous studies in the field, we defined the number of experiments and sample size based on the nature of the sample (whether it is well know or entirely novel genotype, for example), on the variability of the observed phenotypes (larger sample size for more variable phenotypes), and on the complexity of the experimental setup (challenging setups had smaller sample size). The exact sample sizes are described in the figure legends.
Data exclusions	We excluded no datasets from the analyses.
Replication	The phenotypes that we report in this study were reproducible across multiple independent experiments. We performed the experiments in different periods, using different batches, and different experimental and microscopy setups. For the live lightsheet imaging, we performed 7 experiments in btd mutants (total of 50 embryos), 5 experiments in eve mutants (total of 36 embryos), 3 experiments in prd mutants (total of 41 embryos), 6 experiments in slp mutants (total of 39 embryos), 3 experiments in stg mutants (total of 46 embryos), 6 experiments in btd-eve double mutants (total of 35 embryos), and 2 experiments in wildtype embryos (total of 36 embryos). For the germ band cauterization, we performed 6 experiments in btd mutants (total of 10 embryos), 5 experiments in eve mutants (total of 10 embryos), and 8 experiments in wildtype embryos (total of 12 embryos). For the gene expression, the wildtype patterns of btd, eve, and slp were highly consistent across 3 experiments in Drosophila (total of 26 embryos), 3 experiments in Ceratitis (total of 38 embryos), 4 experiments in Anopheles (total of 43 embryos), and 4 experiments in Clogmia (total of 44 embryos). We also obtained consistent patterns of prd expression across 4 experiments in Drosophila (total of 10 embryos) and 1 experiment in Clogmia (total of 20 embryos). Expression patterns in mutant embryos were

	repeatable across 4 independent experiments in slp mutants (total of 30 embryos), 5 experiments in btd mutants (total of 20 embryos), 2 experiments in eve mutants (total of 12 embryos), and 2 experiments in prd mutants (total of 12 embryos).
Randomization	We performed no randomization of samples into experimental groups. However, our control and experimental groups was determined by the zygosity of the embryos (heterozygote or homozygote) which follows genetic inheritance patterns and, therefore, were not known prior or during data acquisition.
Blinding	We performed no blinding of sample for experiments. However, the experimental group (genotype) of the embryos remained unknown during imaging data acquisition and perturbation experiments (laser ablations and cauterizations). We determined the genotypes during data analyses.

Reporting for specific materials, systems and methods

We require information from authors about some types of materials, experimental systems and methods used in many studies. Here, indicate whether each material, system or method listed is relevant to your study. If you are not sure if a list item applies to your research, read the appropriate section before selecting a response.

Materials & experimental systems

n/a	Involved in the study
☒	☐ Antibodies
☒	☐ Eukaryotic cell lines
☒	☐ Palaeontology and archaeology
☐	☒ Animals and other organisms
☒	☐ Clinical data
☒	☐ Dual use research of concern
☒	☐ Plants

Methods

n/a	Involved in the study
☒	☐ ChIP-seq
☒	☐ Flow cytometry
☒	☐ MRI-based neuroimaging

Animals and other research organisms

Policy information about studies involving animals; ARRIVE guidelines recommended for reporting animal research, and Sex and Gender in Research

Laboratory animals	Drosophila melanogaster, Ceratitis capitata, Clogmia albipunctata, and Anopheles stephensi.
Wild animals	The study did not involve wild animals.
Reporting on sex	We did not perform any sex-based analyses as it is not possible to identify the sex of individual flies during embryonic stages.
Field-collected samples	The study did not involve samples collected from the field.
Ethics oversight	No ethical approval is required for research on Drosophila and other dipteran flies.

Note that full information on the approval of the study protocol must also be provided in the manuscript.

Plants

Seed stocks	*Report on the source of all seed stocks or other plant material used. If applicable, state the seed stock centre and catalogue number. If plant specimens were collected from the field, describe the collection location, date and sampling procedures.*
Novel plant genotypes	*Describe the methods by which all novel plant genotypes were produced. This includes those generated by transgenic approaches, gene editing, chemical/radiation-based mutagenesis and hybridization. For transgenic lines, describe the transformation method, the number of independent lines analyzed and the generation upon which experiments were performed. For gene-edited lines, describe the editor used, the endogenous sequence targeted for editing, the targeting guide RNA sequence (if applicable) and how the editor was applied.*
Authentication	*Describe any authentication procedures for each seed stock used or novel genotype generated. Describe any experiments used to assess the effect of a mutation and, where applicable, how potential secondary effects (e.g. second site T-DNA insertions, mosiacism, off-target gene editing) were examined.*
