# Supplementary material for: Patterned invagination prevents mechanical instability during gastrulation
Source: Nature. 2025 Sep 3;646(8085):627–36. doi: 10.1038/s41586-025-09480-3 (PMC12527948; doi:10.1038/s41586-025-09480-3)
Supplement: Supplementary file 4 — Supplementary Videos 1–17 [file 41586_2025_9480_MOESM4_ESM.zip › 2023-10-18195B-s4/TEXT_CF_2025-07-14_main_furrow_supplementary_video_guide.docx]

Patterned invagination prevents mechanical instability during gastrulation

Bruno C. Vellutini^1,✉^, Marina B. Cuenca^1^, Abhijeet Krishna^1,2,3^, Alicja Szałapak^1,2,3^, Carl D. Modes^1,2,3^, and Pavel Tomancak^1,2,3,✉^

^1^ Max Planck Institute of Molecular Cell Biology and Genetics, Dresden, Germany
^2^ Center for Systems Biology Dresden, Dresden, Germany
^3^ Cluster of Excellence Physics of Life, Technische Universität Dresden, Dresden, Germany

^✉^ Correspondence: [Bruno C. Vellutini <vellutini@mpi-cbg.de>](mailto:vellutini@mpi-cbg.de), [Pavel Tomancak <tomancak@mpi-cbg.de>](mailto:tomancak@mpi-cbg.de)

# Supplementary information

## Supplementary information guide

| Title | Description |
| --- | --- |
| Supplementary Video 1 | Reduced apical constriction in *btd* mutants. |
| Supplementary Video 2 | Lateral view of ectopic fold formation in *btd* mutant. |
| Supplementary Video 3 | Lateral view of ectopic fold formation in *eve* mutant. |
| Supplementary Video 4 | Profile view of ectopic fold formation in *btd* mutant. |
| Supplementary Video 5 | Profile view of ectopic fold formation in *eve* mutant. |
| Supplementary Video 6 | Ectopic folding between mitotic domains in *btd* mutant. |
| Supplementary Video 7 | Dynamics of ectopic folding in *btd* mutant. |
| Supplementary Video 8 | Variability of ectopic folding in *btd* mutants. |
| Supplementary Video 9 | Strain rate during ectopic folding in *btd* mutant. |
| Supplementary Video 10 | Lateral view of cephalic furrow formation in *stg* mutants. |
| Supplementary Video 11 | Dorsal view of cephalic furrow formation in *stg* mutants. |
| Supplementary Video 12 | Lateral view of *btd–stg* double mutant. |
| Supplementary Video 13 | Dorsal view of *btd–stg* double mutant. |
| Supplementary Video 14 | Lateral views of germ band cauterization in *eve* embryos. |
| Supplementary Video 15 | Profile views of germ band cauterization in *eve* mutant. |
| Supplementary Video 16 | Profile views of germ band cauterizations in *btd* embryos. |
| Supplementary Video 17 | Lateral view of *slp* mutant. |
